# Localisation and protein-protein interactions of the *Helicobacter pylori* taxis sensor TlpD and their connection to metabolic functions

**DOI:** 10.1038/srep23582

**Published:** 2016-04-05

**Authors:** Wiebke Behrens, Tobias Schweinitzer, Jonathan L. McMurry, Peter C. Loewen, Falk F.R. Buettner, Sarah Menz, Christine Josenhans

**Affiliations:** 1 Institute of Medical Microbiology and Hospital Epidemiology, Hannover Medical School, Hannover, Germany; 2 Department of Molecular & Cellular Biology, Kennesaw State University, Kennesaw, GA, USA; 3 Department of Microbiology, University of Manitoba, Winnipeg, Canada; 4 Institute for Cellular Chemistry, Hannover Medical School, Hannover, Germany; 5 German Center of Infection Research, partner site Hannover-Braunschweig, Germany

## Abstract

The *Helicobacter pylori* energy sensor TlpD determines tactic behaviour under low energy conditions and is important *in vivo*. We explored protein-protein interactions of TlpD and their impact on TlpD localisation and function. Pull-down of tagged TlpD identified protein interaction partners of TlpD, which included the chemotaxis histidine kinase CheAY2, the central metabolic enzyme aconitase (AcnB) and the detoxifying enzyme catalase (KatA). We confirmed that KatA and AcnB physically interact with TlpD. While the TlpD-dependent behavioural response appeared not influenced in the interactor mutants *katA* and *acnB* in steady-state behavioural assays, acetone carboxylase subunit (*acxC*) mutant behaviour was altered. TlpD was localised in a bipolar subcellular pattern in media of high energy. We observed a significant change in TlpD localisation towards the cell body in *cheAY2*-, catalase- or aconitase-deficient bacteria or in bacteria incubated under low energy conditions, including oxidative stress or respiratory inhibition. Inactivation of *tlpD* resulted in an increased sensitivity to iron limitation and oxidative stress and influenced the *H. pylori* transcriptome. Oxidative stress, iron limitation and overexpressing the iron-sulfur repair system *nifSU* altered TlpD-dependent behaviour. We propose that TlpD localisation is instructed by metabolic activity and protein interactions, and its sensory activity is linked to iron-sulfur cluster integrity.

*Helicobacter pylori* colonises about 50% of the world’s population. Chronic colonisation of the human stomach, usually starting in early childhood, results in chronic gastritis. Persistent *H. pylori* infection can also progress to gastric and duodenal ulcer, adenocarcinoma and MALT lymphoma, accounting for more than 500,000 deaths per year worldwide^1^. *H. pylori* is adapted to survive in a challenging niche, the human stomach. Particular challenges of this environment are an acidic luminal pH of about 2^2^, high pepsin activity, which readily inactivates the bacteria^3^, constant mucus turn-over, scarceness of nutrients and metal ions, and prominent host defence mechanisms, for instance oxidative stress^4^,^5^,^6^. These obstacles constantly threaten the survival and persistence of the bacteria. Under these challenging and variable conditions, the majority of *H. pylori* cells is found in close proximity to the epithelial cell layer, within 15 μm above the mucosal surface^7^,^8^, and deep in the stomach glands^9^. Targeted motility and adherence of *H. pylori* to gastric epithelial cells are major factors in colonisation^9^,^10^,^11^,^12^,^13^.

Several *in vivo* studies showed that motility and taxis are of particular importance for *H. pylori* in its niche to navigate to conditions of optimal growth and survival. Non-motile mutants or mutants without a functional chemotaxis system displayed severe colonisation defects or did not colonise at all, which was demonstrated in piglet, mouse and gerbil models^10^,^11^,^13^,^14^. The *H. pylori* chemotaxis system comprises one set of core chemotaxis components^15^,^16^ and four chemotaxis receptors/transducer-like proteins (Tlps)^16^,^17^,^18^,^19^,^20^, TlpA, TlpB, TlpC, TlpD. Cues described so far for chemotactic sensing in *H. pylori* include zinc, nickel^21^, urea^22^,^23^, low pH^18^, bicarbonate, and the amino acid arginine^24^. As in other bacteria, signals sensed by taxis receptors result in a flagellar switch from counterclockwise to clockwise rotation and thereby induce stops, direction changes and backward movements (reversals) of the bacteria, allowing directed motility^25^,^26^. Two of the four *H. pylori* chemotaxis receptors, TlpB and TlpD, have been reported to be linked to energy taxis. Energy taxis can mediate a metabolism-dependent response to intrabacterial energy levels, presumably helping to navigate the bacterium to metabolically favourable environments^13^,^18^,^26^. The *H. pylori* transmembrane receptor TlpB mediates a repellent response to low pH (<pH 3)^18^; low extracellular pH directly influences the proton motive force of the bacteria^27^,^28^, suggesting a connection between TlpB and energy metabolism. TlpB has also been reported to be involved in repellent taxis against the autoinducer metabolite AI-2^29^. We have previously shown that the soluble taxis sensor TlpD, which does not contain transmembrane domains, mediates chemotactic behaviour in response to intrabacterial energy levels in *H. pylori*^13^,^26^. We concluded that TlpD promotes a repellent response to low energy conditions and to inhibitors of the respiratory chain in *H. pylori*. Energy-dependent behaviour was clearly attributed to TlpD in a triple transducer knock-out mutant, and TlpD exerted a dominant effect over the other three sensors under low energy conditions *in vitro*^26^. *In vivo* colonisation experiments in mouse and gerbil models demonstrated that the role of TlpB *in vivo* is variable in mice and relatively minor in the gerbil model^11^,^12^,^18^, while TlpD was very important for the colonisation of the gerbil stomach^13^, and was required for high-level colonisation in the antrum of mice^12^. Our previous inhibitor studies on the function of TlpD suggested that a functional electron transport chain is important for TlpD-dependent sensing and taxis^26^, but more research is clearly required to define the sensing mechanism.

Energy taxis is suggested to play a decisive role for the survival of different bacteria in their niches^30^,^31^. Tlps providing energy-dependent taxis have been described in several species, albeit for most receptors, little is known about the energy sensing mechanisms. Known modes of sensing related to the intrabacterial energy state are diverse^30^. The dedicated *E. coli* energy sensor Aer detects the cellular redox state using a PAS domain and involving FAD cofactor binding, although its precise mode of sensing remains unresolved^27^. A direct function of protein-protein interactions in chemotaxis or energy taxis might merit further investigations, since a direct impact of NADH dehydrogenase on Aer-mediated sensing in *E. coli* was discussed^27^. For the *E. coli* metabolic enzyme ATP synthase, a colocalisation with the flagellar basal body and a direct influence on flagellar rotation was previously reported^32^. Protein-protein interactions involving conformational change might permit quick sensory responses, since shifts of protein conformation can occur rapidly with changing protein activities.

In the present study, we have investigated protein-protein interactions of TlpD and their potential role in TlpD localisation and TlpD-dependent energy sensing of *H. pylori*. We initially identified novel TlpD protein interaction partners using a pull-down approach in *H. pylori*. In addition, we confirmed the direct interaction of TlpD with the enzymes catalase and aconitase. The localisation of the soluble sensor TlpD, which was determined here for the first time, was altered in media of different energy levels and in *cheAY2, acnB* and *katA* mutants. Functional assays using a TlpD wild type strain, overexpression strains, and interactor mutants suggested a role of iron depletion, oxidative stress and iron-sulfur cluster proteins in the TlpD sensing mechanism. The absence of TlpD induced a shift in global transcript activities. These findings indicate a functional connection between the soluble receptor/transducer-like sensor TlpD, its protein-protein interactions and metabolic homeostasis.

## Results

### Protein-protein interactions of *H. pylori* TlpD uncovered by pull-down assay and mass spectrometry

**Figure 1:**
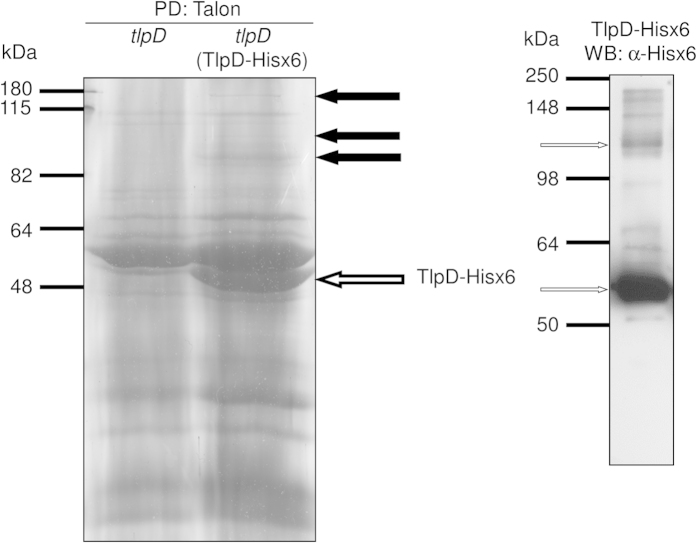
Pull-down of TlpD-Hisx6 with potential protein interaction partners from *H. pylori* N6 cleared cell lysates. Pull-down (PD) precipitates of TlpD-Hisx6 from N6 *tlpD* (TlpD-Hisx6) and N6 *tlpD* (negative control not expressing any TlpD; precipitated using Talon (Cobalt^2+^–coupled matrix)) were separated in 12% SDS gels and stained with Coomassie blue (left panel). Black arrows point at specific higher molecular mass protein bands that were detected only in the pull-down material of *H. pylori* N6 *tlpD* (TlpD-Hisx6) but not in the N6 *tlpD* mutant (control strain). These gel sections, containing differentially detected bands which are numbered 1, 2, 3 from top to bottom (corresponding to numbers in Table 1), were subsequently cut from the gels separately and analysed using mass spectrometry (MS/MS; see Supplementary Methods). The white arrow points at TlpD-Hisx6 monomer. The right panel shows a corresponding Western blot (WB) of the purified TlpD-Hisx6 fraction which was developed using α-Hisx6 antibody. The arrows in the right panel point at TlpD-Hisx6 monomer (lower arrow) and TlpD-Hisx6 dimer (upper arrow).

The transducer-like protein TlpD of *H. pylori* mediates energy tactic behaviour, but its mode of action is still unknown. Its C-terminus is homologous to the signal transducing domain of canonical Tlps^26^, and contains an additional C-terminal zinc-binding domain^33^. The N-terminal domain of TlpD, which is potentially involved in sensing, contains no sequence homology to any known sensing domain in databases (26, and own recent comparison with other proteins in databases). TlpD does not bear strong similarities to other known soluble sensors, e.g. TlpT of *Rhodobacter sphaeroides*^34^,^35^. In order to test the hypothesis that TlpD interacts with other proteins, which may be relevant to its function, we used a pull-down approach to identify potential protein interaction partners of TlpD. We expressed histidine-tagged TlpD (TlpD-Hisx6), which is fully functional in *H. pylori* (Methods), in a *H. pylori* N6 *tlpD::aphA3* insertion mutant from an expression plasmid (pHel2::*tlpD-hisx6*). We then performed pull-down experiments from *H. pylori* grown under standard high energy conditions (blood plates) using Talon affinity beads and visualised TlpD-bound proteins on Coomassie-stained SDS gels. Several protein bands were exclusively detected in the TlpD-Hisx6-expressing strain in comparison to the *tlpD* mutant control (Fig. 1). A prominent TlpD-Hisx6 band and a potential TlpD dimer were detected by anti-His-tag Western blot (Fig. 1). We reproducibly recovered several TlpD-associated protein bands at higher molecular masses (>82 kDa) in three independent pull-down experiments (see black arrows, Fig. 1), which were separately excised and subjected to mass spectrometry analysis. In these analyses (for full results see Table 1), we identified various peptides in each excised band, corresponding to overall six different proteins in addition to TlpD. Peptides for five of the six proteins (chemotaxis histidine kinase CheAY2, aconitate hydratase AcnB, catalase KatA, isoleucyl-tRNA synthetase IleS, general protein secretion translocase SecA, bifunctional RNA polymerase subunit RpoBC), were detected in two independent experimental repeats of the pull-down, respectively. Notably, the only chemotaxis histidine kinase present in *H. pylori,* CheAY2^10^,^36^, which is expected to be interacting with Tlp-orthologous taxis sensors, as previously shown in *E. coli*^37^,^38^,^39^, was identified in both experimental repeats (Table 1). The enzymes AcnB and KatA were also identified in both TlpD pull-down experiments. Due to their direct or indirect importance in metabolic processes, we selected KatA and AcnB for further characterisation in the context of TlpD. We also included the *H. pylori* acetone carboxylase subunit gene (*acxC*) in further functional studies, since AcxC had been identified as a protein that potentially interacts with TlpD in a previous high-throughput Yeast-Two-Hybrid screen^40^ testing for *H. pylori* protein-protein interactions.

### Confirmation of direct *H. pylori* TlpD-KatA and TlpD-AcnB interactions by biochemical interaction studies

**Figure 2:**
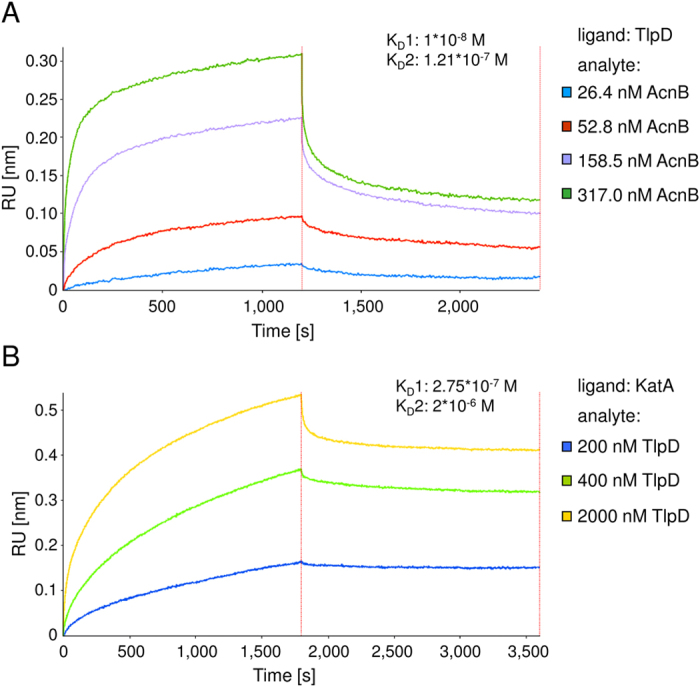
Biochemical analysis of direct interactions of purified *H. pylori* TlpD and AcnB or TlpD and KatA proteins using biolayer interferometry. Recombinantly purified TlpD was tested for direct interactions with purified *H. pylori* AcnB or KatA using biolayer interferometry. While one purified protein (ligand) was coupled to the sensor surface via a hexa-histidine tag or free amine groups (see Supplementary Methods), the second protein (analyte) was applied as a solute in assay buffer. Sensors were dipped into different analyte solutions for 1,200 sec (**A**) or 1,800 sec (**B**), before dissociation was monitored in assay buffer. (**A**) TlpD-AcnB interaction: ligand Hisx6-TlpD (purified from *E. coli*) was immobilised to the sensor surface, while analyte *H. pylori* AcnB (AcnB-V5, purified from *H. pylori*) was applied in assay buffer (Methods and Supplementary Methods) at four different concentrations (26.4 nM, 52.8  nM, 158.5 nM and 317.0 nM). (**B**) TlpD-KatA interaction: immobilised *H. pylori* KatA (recombinant, tag-free, purified from *E. coli*) was coupled on the sensor surface via free amine groups, while analyte TlpD-V5 (purified from *H. pylori*) was supplied diluted in assay buffer at 200 nM, 400 nM and 2,000 nM. Values derived from a ligand-coupled sensor that was dipped in assay buffer only over the full time course of the interaction assay were subtracted as the baseline from the interaction curves.

Our next goal was to verify and characterise the direct interactions of TlpD with KatA and AcnB. We investigated purified recombinant TlpD (see *Methods*) for binding to recombinant active purified KatA^41^ or purified native AcnB (V5-tagged) from *H. pylori*^26^ by biolayer interferometry (BLI), an optical biosensing method that yields kinetic and affinity information in a manner similar to surface plasmon resonance (SPR)^42^. We tested sensor-bound Hisx6-TlpD with different AcnB-V5 concentrations as analyte (Fig. 2A). The data indicated binding and was fit to a two-state parallel binding model which yielded equilibrium dissociation constants K_D_1 of 1*10^−8^ M (fast on, slow off) and K_D_2 of 1.21*10^−7^ M (slow on, fast off) (R^2^ = 0.998). The slow association constant k_on_1 was 2.3*10^4^ M^−1^*s^−1^ and a faster k_on_2 was 2.1*10^5^ M^−1^*s^−1^. Dissociation rate constants k_off_1 and k_off_2 were 2.4*10^−4^ s^−1^ and 2.5*10^−2^ s^−1^, respectively. We also obtained interaction signals for the binding between Hisx6-TlpD (ligand) and KatA (analyte) in the same setting, with a K_D_1 of 3.6 *10^−8^ M and a K_D_2 of 7.8 *10^−9^ M, with a confidence interval R^2^ of 0.96 (data not shown). For direct KatA-TlpD interactions, a two-state parallel binding model also provided the best fit. The KatA-TlpD interaction was also analysed in an inverse experimental setting with KatA as ligand and TlpD (V5-tagged) as analyte (Fig. 2B). We determined K_D_ values of 2.75*10^−7^ M (fast on, slow off) and 2*10^−6^ M (slow on, fast off) in this inverse setting (R^2^ = 0.95). Values of the corresponding rate constants for the association were k_on_1 4.7*10^3^ M^−1^*s^−1^ for the fast and k_on_2 6.1*10^2^ M^−1^*s^−1^ for the slow component (KatA at sensor). For the KatA-TlpD interaction, k_off_ for the two-state model could not be determined due to the very slow off rate. Taken together, we detected *in vitro* reproducible direct, specific interactions of TlpD with AcnB or with KatA, respectively.

### Alterations of subcellular localisation of TlpD in *H. pylori* in the presence or absence of protein interaction partners

We had previously found that the predicted soluble receptor TlpD is partially membrane-associated^26^. The subcellular localisation and potential differential distribution patterns of TlpD, which may be interconnected with its function, were unresolved. To allow for specific localisation studies using immunofluorescence (IF; Supplementary Materials and Methods), we expressed a C-terminally V5-tagged fully functional version of TlpD^26^ in the chromosomal *rdxA* locus (Methods) in the *H. pylori* N6 parental strain.

**Figure 3:**
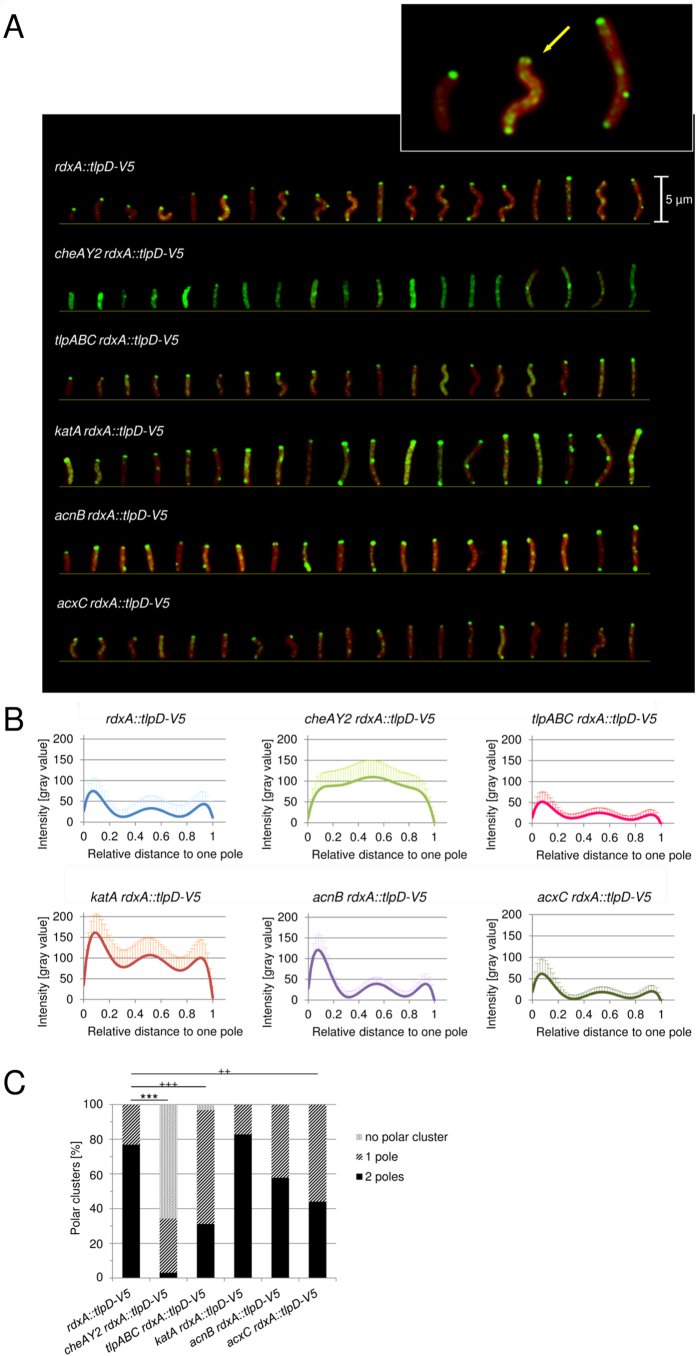
Subcellular localisation of TlpD in *H. pylori* in the presence and absence of potential interaction partners. (**A**) Subcellular localisation of TlpD in intact, permeabilised *H. pylori* cells grown on plates for 20 h (standard high-energy conditions) was imaged by immunofluorescence microscopy (IF; for details of sample preparation see Supplementary Methods). Cells were immediately immersed in fixing agent after harvest. Green: TlpD-V5 was detected using primary mouse α-V5 antibody (1:1,000), combined with secondary α-mouse IgG coupled to Alexa-488 (diluted 1:5,000); Red: SynaptoRed FM4-64 (1:5,000) membrane marker. Specimens were visualised in a Zeiss Apotome fluorescence microscope at a 63-fold lens magnification. A constant exposure time and the same digital image lighting correction functions were used and adjusted equally for all specimens; in each panel, 19 representative cells from one experiment are arranged according to cell length. Arrows point at polar TlpD localisation foci. Inset: three representative images of the N6 *rdxA::tlpD-V5* reference strain were magnified by an additional 4.5 fold. The size bar represents 5 μm. (**B**) Subcellular distribution of TlpD-V5-derived fluorescence intensities (α-V5 MAB) in the *H. pylori* parental strain and corresponding mutant strains deficient in TlpD-interacting proteins AcnB, KatA or AcxC. A *tlpABC* triple transducer mutant, lacking three of the four *H. pylori* Tlps, and a *cheAY2* (chemotaxis null) mutant were used as additional control strains. Growth conditions and sample preparation were the same as in panel A. The fluorescence intensity distribution patterns were quantitated by averaging the pixel intensities in transversal sections (of one pixel length and 8 pixel width) along the longitudinal (x) axes for 15 bacteria from each strain using ImageJ, and then calculating average distribution patterns for all bacteria of each strain (Table 3). The polar and non-polar distributions of TlpD were compared statistically (for details see Supplementary Methods).(**C**) Quantification of polar TlpD clusters per cell for ≥30 bacteria per strain. Clusters at one and both poles were counted visually, and levels of significance were calculated by Fisher’s exact test, for the two conditions polar vs. non-polar clusters (***p ≤ 0.001), and for cluster distribution at 1 vs. 2 poles (^+++^p ≤ 0.001, ^++^p ≤ 0.01).

We initially used IF microscopy to determine the localisation of TlpD in plate-grown bacteria (mid-log phase; growth-dependent high-energy conditions). Immediately upon harvest, bacteria were resuspended in fixing agent to maintain the current energetic conditions. In the reference strain (N6 *rdxA::tlpD-V5*), TlpD was detectable at clearly defined dots at both poles (Fig. 3; Supplementary Fig. S1). In some bacteria, two separate sub-terminal clusters were noted at both sides, close to one pole, corresponding to previous cryotomography results for taxis sensor arrays accumulating on both sides of the flagellar bundle in the related species *Helicobacter hepaticus*^43^. Furthermore, we detected a subfraction of TlpD in more diffuse patches along the whole cell body. Less frequently, dot-like clusters were also present in the bacterial mid-cell region (Fig. 3A). The same distribution of TlpD-V5 was observed in intact cells of strains N6 *rdxA::tlpD-V5* (reference) and N6 *tlpD rdxA::tlpD-V5* which both served as IF positive controls for the expression of functional TlpD^26^ (Methods) (Fig. 3; Supplementary Fig. S1).

Since we hypothesised that interactions of TlpD with other proteins may have consequences for TlpD localisation and/or function, we constructed isogenic allelic exchange mutants in *H. pylori* N6 (wild-type strain) and in the *H. pylori* N6 *rdxA::tlpD-V5* parental strain (reference to test TlpD localisation), each deficient in one of the genes encoding the *H. pylori* TlpD interaction partners, *cheAY2, acnB, katA,* and the potential interaction partner *acxC*^40^ (Table 2). Except for the *acnB* mutants, which had a severe growth defect, none of the other mutants showed an obvious growth deficiency *in vitro* or other gross phenotypical alterations in comparison to the parental strains. All the mutants also displayed normal morphology in transmission electron microscopy (not shown).

We then used IF microscopy to determine the localisation of TlpD in these strains (plate-grown, same conditions as for the N6 *rdxA::tlpD-V5* parental strain). We noted obvious differences between the parental strain and its *cheAY2* and *katA* mutants. In the former, TlpD appeared to be almost lost at the polar localisation, while, in the latter, relatively more TlpD was observed throughout the cell body (Fig. 3). For the more precise comparison of TlpD localisation between the different interactor mutants, we quantitated signal intensities in IF (Fig. 3; Supplementary Figs S1 and S2) and Western blots (Supplementary Fig. S3), using densitometry. First of all, we verified that TlpD-V5 was present at similar levels in all tested strains, which was indeed the case (Supplementary Fig. S3). We subsequently quantitated the polar TlpD clusters and the average fluorescence intensities of TlpD at different subcellular locations (polar, non-polar; Methods). We compared the localisation patterns for each cell by enumerating the polar clusters, and by averaging multiple cells of each strain followed by statistical evaluation (uni- versus bipolar or no polar clusters; Fig. 3C; Methods) between the reference strains and all interactor mutants. Furthermore, we compared the fluorescence intensities in the cell body versus the polar regions (Fig. 3B; Table 3). The mutant in the central chemotaxis histidine kinase gene *cheAY2*, a taxis null mutant, exhibited a significant delocalisation of TlpD away from both poles (Fig. 3A–C; Table 3) and a loss of polar TlpD in almost all cells (see Table 3 for percent values, Fig. 3A–C). Even in the virtual absence of polar clusters in the *cheAY2* mutant, TlpD was still detectable in diffuse clusters along the entire cell body (Fig. 3A; Supplementary Figure S2). The triple transducer knockout strain *tlpABC* that we used as an additional control strain, which contains TlpD as the only Tlp sensor, partially lost polar TlpD. In the latter strain, we detected TlpD predominantly at one pole in contrast to the bipolar distribution in the parental strain (Fig. 3A–C). Remarkably, the *katA* mutant displayed a significantly increased TlpD signal in the cell body over the polar regions in comparison to the parental strain (Fig. 3A–C; Table 3). In contrast, the *acxC* mutant showed a less obvious but still significant shift of relative TlpD intensity towards polarly localised signal, with a preferentially unipolar distribution (Fig. 3A–C). In contrast, the *acnB* mutant did not exhibit a significant change in subcellular TlpD localisation under these standard incubation conditions (20 h plate-grown bacteria, directly resupended in fixing agent; Fig. 3A–C; Table 3).

To refine the *in situ* characterisation of TlpD subcellular localisation, we used Western blots of whole cell lysates of the *katA, acnB*, and *acxC* mutant strains and control strains fractionated into soluble and insoluble (membrane-enriched) fractions. TlpD was quantitated in both fractions of each strain by densitometry (Supplementary Fig. S3). In all tested mutants, we detected TlpD in both the insoluble and soluble protein fractions without any significant differences in TlpD amounts or distribution between the strains (Supplementary Fig. S3; approximately 5% TlpD in insoluble, 95% in soluble fraction). Interestingly, higher molecular mass (MM) protein bands containing TlpD were similarly present in wild type and mutants even with denaturing SDS gels in the presence of reducing agent (Supplementary Fig. S3; Methods). In control strains, harbouring single knock-out alleles in *acnB, katA* or *acxC*, which did not contain an additional *tlpD-V5* allele in the *rdxA* locus, a similar distribution of TlpD between the soluble and insoluble bacterial fractions was also detected in all strains (not shown).

### Inactivation of TlpD interaction partners KatA, AcnB and AcxC maintains TlpD-dependent steady-state behaviour

We hypothesised that potential TlpD interaction partners can be, either directly or indirectly, involved in energy taxis, and, in this context, might influence TlpD-mediated energy-dependent behaviour. In order to test this hypothesis, *katA, acnB, acx*C, and *cheAY2* isogenic mutant strains expressing wild type TlpD were subjected to steady-state behavioural assays (single cell tracking assays) in liquid media under conditions of low intrabacterial energy, under which TlpD is functionally dominant and the TlpD-dependent phenotype well characterised^26^. Under these conditions, TlpD mediates a high number of stops and direction changes^13^,^26^.

**Figure 4:**
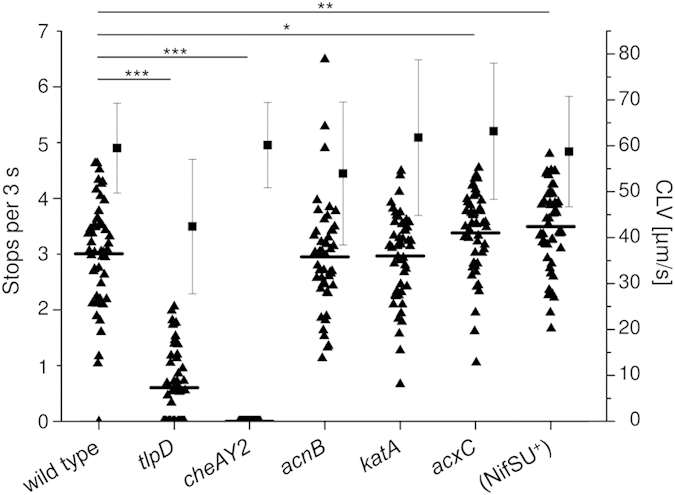
Characterisation of behavioural patterns for different *H. pylori* mutants in genes coding for TlpD-interacting proteins (swim assay). Behavioural patterns of *H. pylori* were determined by tracking assays using 50 bacteria per strain under previously determined steady state conditions of low bacterial energy yield^13^,^26^ in RPMI 1640 + 3% FCS at 37 °C and ambient air enriched with 5% CO_2_. Bacteria were collected from blood agar plates after 20 h of growth and resuspended in the liquid medium of low energy yield. Under these conditions, stopping frequency is dominated by TlpD^26^. Stops per three seconds (black triangles with mean) and curvilinear velocity (CLV; average with standard deviation as black squares with whiskers) were quantified for the different mutants. Significance values for differences between the strains were calculated by Student’s *t* test.

*katA* and *acnB* mutants did not show a significantly altered swimming speed or motile behaviour in comparison to the parental strain, indicating that the respective proteins do not play a role in TlpD-mediated behaviour in these assay settings (Fig. 4). The *acxC* mutant showed a slight but statistically significant upshift in TlpD-mediated steady-state behaviour (increased stops). The *cheAY2* mutant, as expected, was uniformly smooth-swimming under all incubation conditions, which is a known chemotaxis null phenotype as described before^26^. We had already previously assayed *tlpABC* mutants^26^, which exhibited a reduced TlpD-dependent stopping behaviour in comparison to the parental strain.

### *H. pylori tlpD* mutants are more susceptible towards iron depletion and oxidative stress and show an altered transcriptome

Iron-limiting conditions and oxidative stress are two major factors characteristic of the *H. pylori in vivo* environment^13^. The host mucosal milieu is poor in iron, due to strong iron-sequestering factors of mammalian cells, and the proinflammatory host innate immune response provides strong oxidative stressors. Previous functional assays^13^,^26^ had suggested that intrabacterial energy and oxidative stress, which impacts on central energy metabolism via central iron-sulfur proteins^44^,^45^, both may play a role in the TlpD-mediated sensing process. Taken together with our previous, unexpected observation that the *tlpD* mutant has an increased susceptibility to oxidative stress^13^ these results strengthened a link between TlpD and iron acquisition or iron-sulfur homeostasis.

Supplementing our previous results of paraquat treatment^13^, we specifically investigated the impact of iron depletion on the growth of *H. pylori* N6 wild type, *tlpD* mutant and the complementation strain, using 2,2-dipyridyl (2,2-DP) in agar diffusion assays. *tlpD* mutants in two *H. pylori* strains, N6 and HP87P7, were more sensitive to iron depletion by 2,2-DP (significantly increased zone of growth inhibition) than the respective wild type (Table 4), similarly as previously found in the presence of paraquat^13^. The phenotype was reconstituted back to wild type by *tlpD* complementation in the chromosomal *rdxA* locus (Table 4), which confirmed that the phenotype was indeed mediated by the lack of TlpD.

To investigate whether a change in gene regulation may be one cause of different susceptibility of the *tlpD* mutant to these conditions, we performed genome-wide microarray transcriptome analysis of the *tlpD* mutant in comparison to the *H. pylori* N6 parental strain. Overall, 68 genes were found either at >2-fold higher (20) or <2-fold lower transcript levels (48) in the *tlpD* mutant in comparison to the wild type strain (Supplementary Tables S1 and S2). Several transcripts coding for iron-provisioning proteins or iron-sulfur proteins were detected at increased amounts in the *tlpD* mutant, including the iron-sulfur biosynthesis protein operon NifSU (HP0220-HP0221)^46^,^47^. An increase in *nifU* transcript was confirmed by qRT-PCR in *tlpD* mutants of two strains (Supplementary Fig. S4A,B).

### TlpD-dependent steady-state behaviour of *H. pylori* is altered under conditions of low iron or high oxidative stress, and by NifSU supplementation

**Figure 5:**
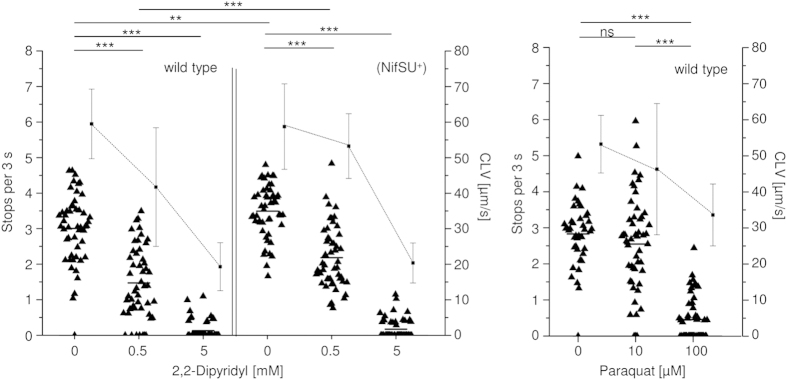
Characterisation of *H. pylori* motility patterns under conditions of iron depletion or oxidative stress. Tracking assays were performed under steady state conditions as described before (see also legend to Fig. 4; low-energy liquid medium RPMI 1640 with 3% FCS). The iron chelator 2,2-dipyridyl or the oxidative stress-inducer paraquat were added to the medium at indicated concentrations, and motile steady-state behaviour was analysed by single cell tracking and quantitated by stop frequencies and curvilinear velocity (CLV) (black squares with whiskers). For each condition, at least 50 cells were traced (black triangles with mean). Significance of differences was calculated using Student’s *t* test. ***p ≤ 0.001; **p ≤ 0.01 ns is non-significant. N6 (NifSU^+^) is a strain expressing the *H. pylori nifSU* genes from a plasmid (Results and Supplementary Methods).

We performed behavioural assays in the presence of paraquat (10 μM and 100 μM) or iron depletion by 2,2-DP (0.5 mM and 5 mM) for N6 wild type, *acnB* and *katA* mutants. The assays dose-dependently revealed a reduced TlpD-mediated stopping frequency for all tested strains, similarly with paraquat or 2,2-DP (Fig. 5, results for the parental strain; mutants not shown). Almost no stops were observed at the higher concentrations of 100 μM paraquat or 5 mM 2,2-DP (Fig. 5), respectively, suggesting a comparable effect of both conditions on behaviour.

The *tlpD* mutant, as shown above, was more sensitive than the wild type to oxidative and iron depletion-related stress, although the transcriptome analyses revealed an increase in transcript amounts of some genes, including the *nifSU* operon, which potentially counteract these environmental stressors (Supplementary Fig. S4). In order to test the hypothesis that iron-sulfur cluster damage or repair may exert an influence on energy-dependent sensing by TlpD, we targeted the essential *nifSU* genes (HP0220-HP0221). We introduced an expression plasmid for NifSU into *H. pylori* N6, providing additional gene copies under the control of the intrinsic promoter. This strain, N6 (pHel2::*nifSU*) (N6 (pCJ1350), in short: N6 (NifSU^+^)) was viable and showed no growth changes, but had an elevated cellular ATP content (not shown; quantitated as one marker for metabolic activity as described in^26^) compared to the parental strain. When we tested the N6 (NifSU^+^) strain for swimming behaviour, TlpD-mediated behaviour was reproducibly altered in comparison to the parental strain (Figs. 4 and 5). Bacterial stopping frequencies were significantly increased in comparison to the parental strain, suggesting that a reactivity threshold for TlpD-dependent responses was shifted in this strain. As expected, the resistance of the N6 (NifSU^+^) strain towards low-iron stress (iron depletion by 2,2-DP supplementation) was enhanced in comparison to the parental strain (Supplementary Fig. S5).

### TlpD shows altered subcellular localisation in *H. pylori* under incubation conditions of different energy yield, respiratory inhibition and oxidative stress

Previously, we were able to match the differences in ATP production by *H. pylori* in different media to the TlpD-mediated behavioural responses: for instance, BHI containing fetal calf serum (FCS) provided higher ATP levels to *H. pylori*, while ATP levels were markedly lower in the defined medium RPMI 1640 containing the same amount of serum^26^; concomitantly, TlpD-mediated responses were prominent in the low-energy medium, while TlpD-mediated responses were minor in the higher energy medium^26^. In addition, a metabolic inhibitor of the electron transport chain, myxothiazol, induced a TlpD-mediated repellent response and a concentration-dependent change in steady-state swimming behaviour^26^. In order to verify whether the subcellular distribution of TlpD was associated with the energy content of the growth medium, we shifted *H. pylori* from plate-grown conditions to the two different liquid media of higher (BHI-FCS) and lower (RPMI-FCS) energy yield^26^. We incubated the bacteria for a short period of 20 min in these liquid media, pelleted them at low temperature, and immediately resuspended them in fixing agent to preserve their energetic state. We also included additional conditions, inducing alterations in the bacterial energy status in energy-rich liquid medium (BHI-FCS) by direct (myxothiazol added at 10 μM; 26) or indirect metabolic inhibition (oxidative stress by paraquat supplementation, at 10 μM concentration). Under these different energy repletion or depletion conditions, we compared the relative localisation of TlpD by the quantitation of localised fluorescence intensities in the above strains expressing TlpD-V5 from the chromosomal *rdxA* locus.

**Figure 6:**
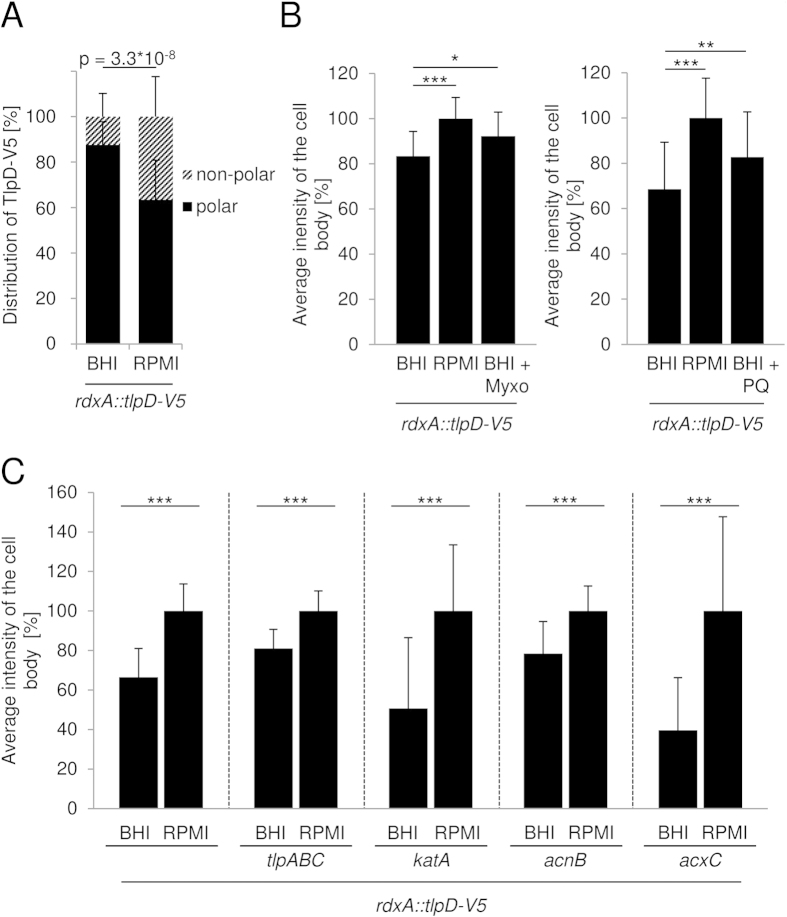
Subcellular localisation of *H. pylori* TlpD under different energetic conditions. (**A**) TlpD-V5 localisation at bacterial poles and cell body was quantitated by immunofluorescence (IF) and compared for N6 *rdxA*::*tlpD-V5* (reference strain) in BHI-yeast, 3% FCS (BHI, high energy yield^26^) and RPMI 1640, 3% FCS (RPMI, low energy yield^26^). For IF quantitation, 20 h plate-grown bacteria were incubated in media for 20 min, then immediately fixed and immunolabelled (Supplementary Methods). Total cell intensities were set to 100%; polar and cell body pixel intensities are depicted in percent of total intensities. p-value indicates significant difference between conditions for poles or cell body. (**B**) TlpD-derived intensities for the cell body only were determined for the reference strain to detect energy-dependent differences in non-polar TlpD. Incubation conditions were: BHI, RPMI, BHI and myxothiazol^13^ (Myxo, 10 μM for 10 min; left panel, for respiratory inhibition), or BHI and paraquat (PQ, 10 μM for 30 min; right panel for oxidative stress). Myxothiazol or PQ was added after 20 min equilibration in BHI. The fluorescence intensity of the sum of all pixels in the cell body without the poles was first calculated in percent of the total cell pixel intensities added up for each strain; subsequently, cell body-associated intensities in RPMI were set to 100%. Fluorescence intensities of the bacterial cell body omitting the poles were averaged for 30 bacteria under each condition (Supplementary Methods). (**C**) TlpD localisation in the cell body in the reference strain and the corresponding *acnB, katA, tlpABC, acxC* mutants was determined under two energetic conditions. 20 h plate-grown bacteria were resuspended in liquid media RPMI or BHI for 20 min. Cell body intensities in RPMI were calculated as in B and set to 100% (right bar for each strain), with normalised relative values in percent for the BHI condition (Supplementary Methods). For all panels and settings, mean and standard deviations of TlpD-derived intensities averaged for 30 bacteria are depicted. IF images were taken at 63-fold magnification. Poles and cell body regions were defined as in Table 3. Significance of differences was determined using Student’s *t* test (***p ≤ 0.001, **p ≤ 0.01, *p<= 0.05).

Remarkably, in the reference strain (*H. pylori* N6 *rdxA::tlpD*-*V5*) incubated in liquid media of different energy yield, we observed a differential subcellular distribution of IF-labelled TlpD between high energy and low energy medium. In fresh, high-nutrient growth medium in the parental strain, TlpD was predominantly polar (87.6% polar intensity versus 12.4% non-polar, standard error 10.2%; Fig. 6A; Supplementary Fig. S6A). In energy-limited medium at the same time point post incubation (20 min), this relative distribution was significantly shifted towards the cell body (63.5% polar versus 36.5% non-polar TlpD intensity, standard error 17.4%; Fig. 6A, Supplementary Fig. S6A). Likewise, when oxidative stress was applied in high-energy medium using paraquat, or metabolic stress in the presence of the respiratory inhibitor myxothiazol, a significantly larger fraction of total TlpD in the reference strain was non-polar as compared to the energy-rich medium. This was reflected by significantly higher relative TlpD intensities in the cell body under these conditions of metabolic stress (Fig. 6B; Supplementary Fig. S6A). When we tested the TlpD interactor mutants (*acnB, katA, acxC*) under the same conditions in two different media, they also showed a statistically significant enrichment of TlpD in the cell body in energy-limited medium versus high-energy liquid medium (Fig. 6C). The effect of short-term incubation in liquid media was particularly pronounced for the *acnB* mutant, which, in comparison to the parental strain, showed a significant redistribution of TlpD towards the cell body even in the high-energy liquid medium (Supplementary Fig. S6C). The *katA* mutant, which already differed from the parental strain under standard growth conditions (relative TlpD intensity higher in the cell body), also showed a significant difference in relative TlpD intensity in the cell body between high-energy and low-energy liquid medium (Figs. 3 and 6C).

## Discussion

### Energy sensor TlpD interacts with several non-chemotaxis proteins, including catalase and aconitase

**Figure 7:**
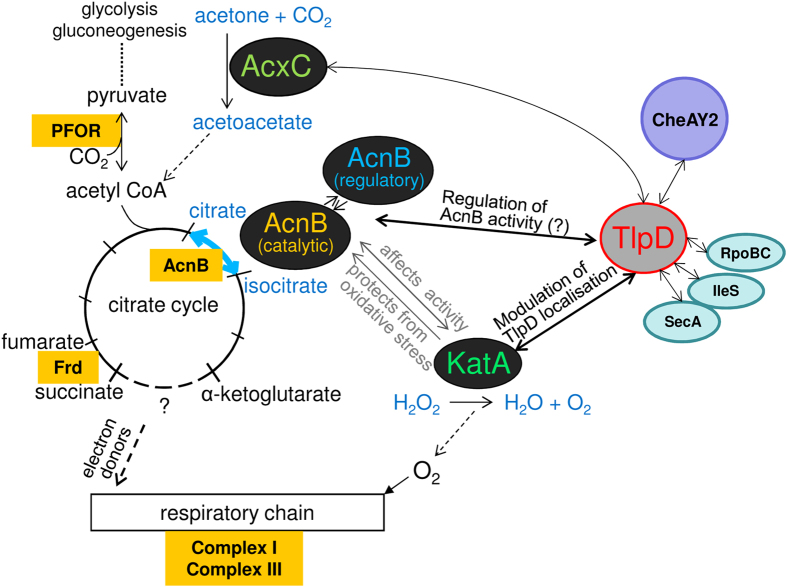
Model of protein-protein interactions of the *H. pylori* energy sensor TlpD and their connection to metabolism and gene regulation. Several novel TlpD protein-protein interactions were identified in the present study (KatA, AcnB, CheAY2, IleS, RpoBC, SecA). This model depicts how some of these proteins, jointly with TlpD, integrate into the network of *H. pylori* metabolism and energy-related functions, which may feed back into TlpD-mediated energy sensing. AcnB is a bifunctional enzyme of the TCA cycle, converting citrate into isocitrate and vice versa. In *H. pylori*, microaerobic metabolism is thought to favour the generation of citrate by AcnB, feeding into CO_2_ fixation and gluconeogenesis^53^. KatA^80^ detoxifies H_2_O_2_ and oxygen radicals, and might thereby protect metabolic enzymes containing iron-sulfur clusters such as AcnB. The putative interactor AcxC is a subunit of acetone carboxylase^54^, which synthesises acetoacetate, an important precursor of the gluconeogenesis and TCA cofactor acetyl-CoA. Pyruvate-ferredoxin oxidoreductase (PFOR), an iron-sulfur enzyme essential for the microaerobic, capnophilic metabolism of *H. pylori*^51^,^52^, can also govern the direction of metabolic flow in *H. pylori* from the citrate cycle to gluconeogenesis by CO_2_ fixation and pyruvate generation. By repleting acetyl-CoA, acetone carboxylase also supplements the PFOR reaction and gluconeogenesis. Under conditions of oxidative stress, AcnB switches between a catalytic and a regulatory function^75^. TlpD may interfere with AcnB activity and influence regulatory functions of AcnB under stress conditions. KatA possibly protects AcnB from oxidative stress to maintain AcnB catalytic function under oxidative conditions. Proteins containing iron-sulfur clusters^46^ which are integrated into this part of the metabolic network and may influence TlpD sensing under oxidative or metabolic stress (AcnB, fumarate reductase Frd, PFOR, and subunits of the respiratory complexes I and III) are highlighted by orange colour. Verified interactions between proteins are indicated by bold arrows. Additional potential interactors of TlpD whose direct interaction has not yet been clarified (SecA, IleS, RpoBC) are depicted for completeness.

A Yeast-Two-Hybrid protein-protein interaction study of *H. pylori* proteins had suggested before that *H. pylori* TlpD is able to interact with other bacterial proteins^40^, providing one motivation for the current study. Protein-protein interactions involving conformational change may be a means to modulate TlpD-related functions, which might be a key to understand mechanisms of TlpD function. Interactions of chemoreceptors with other proteins may be involved in the mechanism of sensing and also in soluble taxis sensor localisation, although few examples have been reported. For instance, interaction of the soluble cytoplasmic MCP of *Rhodobacter sphaeroides*, TlpT, with a novel type of partitioning protein, PpfA, determines the subcellular positioning and cluster segregation of the cytoplasmic sensor upon cell division^34^. Using a pull-down approach against Hisx6-tagged TlpD combined with mass spectrometry of specifically enriched protein bands from *H. pylori* lysates, we identified six novel potential protein interaction partners of TlpD, CheAY2, AcnB, KatA, IleS, SecA, and RpoBC. The mass spectrometry analyses did not reveal a dedicated partitioning protein interacting with TlpD. Since the analysis was restricted to the fraction of TlpD that became soluble during the lysis procedure, we might have missed yet other potential interactors. Among the novel TlpD-bound proteins was CheAY2, the histidine kinase propagating signals from Tlp sensors towards the flagellar basal body^10^. In *E. coli*, the current concept for MCP interactions with the chemotaxis core proteins is that MCPs can dock with CheW^25^ or with CheA directly, using the CheA P5 domain^37^,^39^,^48^. Although we have not experimentally followed up specific binding between these two proteins, TlpD and CheAY2, in the current study, the detection of the intrabacterial TlpD-CheAY2 interaction in *H. pylori* is therefore highly plausible and indicates that the pull-down approach was able to reveal true *in-vivo* interactions. Interestingly, two proteins directly involved in bacterial metabolic functions were also identified as TlpD interactors, aconitate hydratase (AcnB^49^, a key enzyme in the central metabolism and tricarboxylic acid [TCA] cycle, containing one iron-sulfur cluster per subunit) and catalase (KatA^41^,^50^, crucial in cellular detoxification of hydrogen peroxide and oxygen radicals). KatA is the only catalase in *H. pylori*^50^. The bidirectional catalytic reaction mediated by AcnB in the TCA cycle (Fig. 7) is considered to be central to two major functions of the capnophilic *H. pylori* TCA cycle, first by providing substrates for CO_2_ fixation and glycogenic pyruvate assimilation by the pyruvate-ferredoxin oxidoreductase (PFOR) enzyme, upstream of citrate^51^,^52^; and second, by generating electron donors (NADPH^+^) for the respiratory chain through the TCA enzymes downstream of isocitrate^53^ (see Fig. 7 for AcnB function and integration into metabolic circuitry). *H. pylori acnB*^49^ and *katA*^50^ mutants as well as another mutant in the *acx* operon (*acxB;* 54) were described before. All three genes were non-essential *in vitro. acnB, katA* and *acx(B)* mutants displayed a severe colonisation-deficient phenotype in mice^54^,^55^,^49^, underlining their important role *in vivo*.

We verified that KatA and AcnB can act as direct physical binding partners for TlpD *in vitro* using biolayer interferometry. Both proteins interacted with TlpD with a K_D_ in the nanomolar range. A two-state parallel binding model was best fitting the data for both interactions (TlpD-KatA and TlpD-AcnB). The determined complexity of the binding curves may reflect particularities of the BLI method such as presentation of the ligand to the analyte, but may also be suggestive of the so far unknown mechanisms of the biological interactions. Interestingly, an earlier study which used a Strep-tag pull-down approach to identify large protein complexes in *H. pylori* identified both AcnB and KatA as components of a large complex involving urease subunits^28^. This earlier publication already provided an indication that both AcnB and KatA jointly can be part of one single large protein complex, involved in metabolic homeostasis. Three other intrabacterial protein interactions of TlpD identified by the pull-down were to isoleucyl-tRNA synthetase IleS, to the bifunctional RNA polymerase subunit RpoBC, and to the protein translocase-ATPase SecA. These three additional interactions remain to be characterised with respect to direct or indirect binding to TlpD, regulatory function, TlpD activity, or TlpD subcellular positioning.

For the functional characterisation of TlpD, we included a third metabolism-related protein, AcxC, in addition to AcnB and KatA. AcxC (HP0697, encoding an acetone carboxylase subunit^54^), was identified earlier as a potentially TlpD-interacting protein in a large-scale *H. pylori* Yeast-Two-Hybrid interaction study^40^. However, we did not perform direct binding analyses for AcxC to confirm this proposed interaction, and it is therefore not excluded that this candidate was a false positive.

### TlpD localisation in *H. pylori* is influenced by protein interactions, cellular energy status and oxidative stress

The subcellular localisation and distribution patterns of TlpD were previously unresolved. In order to gain better insight into TlpD localisation, function, or potential mechanisms of TlpD energy sensing, we investigated the subcellular localisation of the soluble sensor TlpD under different conditions. While we had previously obtained a preliminary result of membrane-associated localisation of TlpD using an expression plasmid which introduced a gene copy effect^26^, we were now able by chromosomal integration to obtain more accurate data on the intracellular TlpD localisation *in situ* which also permitted to dissect subtle differences. Under standard culture conditions *in situ*, we found TlpD to be localised both focally at the cell poles and in diffuse clusters along the cell body. Although TlpD was predicted to be a soluble sensor by sequence, it was invariably present in similar proportions in the insoluble as well as soluble fractions in all fractionated lysates of mutants and parental strain.

We hypothesised that TlpD localisation might be connected to its function. Therefore, we addressed TlpD localisation in *H. pylori* isogenic mutants lacking TlpD-interacting proteins and in the context of metabolic and oxidative stress. Some of the newly identified protein interaction partners had an influence on TlpD subcellular localisation. Inactivation of the *H. pylori* core chemotaxis kinase CheAY2^10^ (taxis null phenotype) had the strongest impact and led to an almost complete loss of TlpD at the polar localisation foci. Both, TlpD polar localisation and a loss of polar accumulation in the absence of CheAY2, were not expected, since TlpD would not necessarily have to interact at the poles with CheAY2 to be active. However, we interpret this localisation as a result of the interaction of TlpD with a fraction of CheAY2 at the poles, which is supposed to be maintained there, for instance via direct interaction with the canonical membrane-bound chemoreceptors^37^,^38^,^48^,^56^. Very recently, CheAY2 was indeed reported in *H. pylori* to localise to the bacterial poles, mediated by the presence of the Tlps^57^, which is consistent with our interpretation. In the same study, the authors also detected a predominantly bipolar localisation of the *H. pylori* Tlp family, with some signal distant from the poles. TlpD localisation on its own *in situ* was not addressed in the other study; however, the results match our findings that both bipolar and non-polar TlpD clusters exist in the *H. pylori* cell.

Although the three transmembrane Tlp sensors of *H. pylori* are not essential for TlpD function, TlpD-mediated behaviour was significantly diminished in *tlpABC* triple mutants which express TlpD as the only taxis sensor, as shown before^26^. When testing the *tlpABC* mutants for TlpD localisation, we demonstrated that they retained TlpD at one pole, but virtually lost the bipolar localisation characteristic of the parental strain. In accordance with other recent data on the subcellular localisation of chemotaxis components in *H. pylori*^57^, it is plausible to speculate that TlpD is retained preferentially at one pole by associating with the polarly localised transmembrane Tlps^57^, while it may be kept at the other pole by so far unknown interactions possibly involving CheAY2. With regard to a role in TlpD localisation of the additional novel TlpD interactors identified by pull-down, we also detected a clear alteration in bacterial subcellular localisation of TlpD in *katA* mutants, where a significant shift of TlpD towards the cell body occurred under standard incubation conditions. In *acxC* mutants, TlpD showed a shift towards a preferentially unipolar localisation, similar to the triple-transducer mutant. We currently have no mechanistic explanation for the latter result.

With respect to conditions that impact on metabolic activities, we have shown here that in the parental strain TlpD resided primarily at both poles (more than 80% of total TlpD-derived fluorescence signal) under defined conditions of high nutrient availability, under which TlpD net activity is minor^26^. Under nutrient-limited conditions, which favour a dominance of TlpD function over the other *H. pylori* Tlps^26^, a significant proportion of TlpD was delocalised towards the cell body. The same effect occurred under the influence of metabolic inhibitors or under conditions of oxidative stress. This raised the question whether this was an effect of protein redistribution, indicating protein dynamics, or of *de novo* synthesis of TlpD, possibly starting at non-polar sites. This open question will need to be addressed in detail, for example by conducting the experiments in the presence of protein synthesis inhibitors. Interestingly, a metabolism-dependent localisation of a soluble energy taxis receptor was described previously in a seminal study for AerC, a PAS-domain-containing taxis sensor in *Azospirillum brasilense*^58^. AerC distributed differentially, either polar or disperse, in different states of bacterial nitrogen fixing activity^58^. A more recent study concluded that metabolic conditions can guide the dynamic positioning of a respiratory membrane complex (nitrate reductase) in *E. coli* to shift between the poles (anoxic) and the total circumference of the bacteria (oxic conditions)^59^. The latter study also revealed that protein *de novo* synthesis was involved in the dynamics of the respiratory complex when the bacteria were shifted from oxic to anoxic conditions, but not vice versa^59^. It was not clarified whether the contribution of protein synthesis was direct or indirect in the process.

What can be the mechanisms responsible for TlpD localisation differences in certain mutants? For one, protein-protein interactions can directly influence localisation. We assume that in *cheAY2* and triple transducer mutants, shifts in TlpD localisation away from one or both poles are most likely mediated by direct interactions within heterogeneous protein complexes, as explained above. However, we have no unambiguous explanation for the significant alterations in TlpD localisation in *katA* and *acnB* mutants versus the parental strain. While a role of the direct protein interactions is possible, an alternative scenario which could explain some changes in TlpD localisation is a general shift of metabolic activities in the mutant bacteria. A strong contribution of the latter scenario appears most probable for the *acnB* mutant, which showed a severe growth defect and a reduced metabolic activity in several assays (own unpublished data). In accordance, the loss of AcnB appeared to mediate a significant difference in subcellular distribution of TlpD towards the cell body only in high-energy liquid medium with respect to the parental strain. However, in the *katA* mutant, while we did not observe a change in growth, ATP content, respiratory activity, or altered TlpD-mediated behavioural response, TlpD was significantly enriched in the cell body versus the poles. Therefore, we currently assume a direct influence of KatA on TlpD localisation. The localisation differences suggest that KatA, of which a significant insoluble portion exists in *H. pylori* according to our present results (Fig. S3), may contribute to retaining TlpD at the poles under conditions of high energy, in which the contribution of TlpD to the net bacterial behaviour was minor^26^. We still have to clarify whether TlpD performs its function at any particular subcellular localisation, i.e. whether it might be more active at the non-polar location versus the poles.

In other bacterial species, dedicated energy harvesting complexes of the respiratory chain were located in close proximity to the basal body of the flagellar apparatus^32^. Whether TlpD incorporates into similar complexes will need to be further investigated. As AcnB catalytic activity was reduced in *H. pylori katA* mutants (own unpublished data), KatA might provide a functional connection between TlpD and AcnB, which needs to be further explored (see also model in Fig. 7). Taken together, our data hint toward a functional and possibly dynamic, physical connection between metabolically relevant enzymes such as KatA, AcnB, and other energy-generating complexes in *H. pylori* with components of the chemotaxis and motility machineries.

### TlpD-related behaviour remains unchanged by deficiency in the interactors AcnB or KatA, but is modulated by oxidative stress, iron depletion and iron-sulfur homeostasis via NifSU

In addition to localisation, we hypothesised that AcnB, KatA or other potentially interacting proteins, by catalytic activity status or conformational alterations, might influence the energy-dependent taxis function of TlpD. We phenotypically characterised mutants in the potential interaction partners AcnB, KatA and AcxC for changes in their behavioural repertoire linked to the TlpD sensor. Since our microarray analyses revealed compensatory regulation in the absence of functional TlpD, for instance a transcript increase of the iron-sulfur cluster maintenance gene *nifU*^46^, we also included the characterisation of NifSU overexpression with respect to TlpD function.

We did not observe a significant modulation of *H. pylori* steady-state motile behaviour when analysing the interactor mutants *katA* or *acnB*, despite the fact that TlpD localisation, in particular in the *katA* mutant, was changed. Differential influences of mutants on TlpD localisation versus function are not excluded by prior data, and future investigation will have to take other interacting proteins, protein complexes and alternative incubation conditions into account. The *tlpABC* triple mutant had a reduced TlpD response activity as noted previously^26^. Interestingly, AcxC deficiency and overexpression of the NifSU iron-sulfur cluster biogenesis proteins also led to a significant modulation in TlpD-mediated steady-state behaviour. Since the NifSU gene products in *H. pylori* are involved in the maintenance and repair of iron-sulfur cluster proteins such as aconitase, PFOR, fumarate reductase, hydrogenase, ferredoxins and other enzymes feeding into or directly involved in the *H. pylori* respiratory chain^46^,^47^, they could provide one missing functional link between intrabacterial energy generation, redox homeostasis and energy taxis. Such a link was previously suggested by experiments conducted with inhibitors of the respiratory chain which hampered TlpD function and induced a repellent response^26^. TlpD-dependent stopping frequency was augmented by NifSU. This result was correlated to the inverse effect of iron depletion (2,2-dipyridyl) and increased oxidative stress (paraquat) which both diminished the behavioural response by TlpD. It is a possible scenario that NifSU supplementation mediates a general metabolic shift in the bacteria by acting on a host of important metabolic iron-sulfur-cluster enzymes simultaneously and thus might influence TlpD-dependent sensing indirectly.

Although the *tlpD*-deficient strain was more sensitive than its parent against iron depletion or oxidative stress, *H. pylori* in general showed a relatively weak sensitivity. This can be explained by the technical limitations of the used test system (agar diffusion) and by strong redundancies in the bacterial detoxifying systems in *H. pylori* which counteract iron and oxidative stresses^60^,^61^.

The TlpD-dependent behavioural phenotype^13^,^26^ was very robust in different *H. pylori* mutants. Except for the chemotaxis null mutants *cheAY2*^10^,^26^, *cheW*^62^,^63^, *cheY*^10^,^62^ and mutants in *tlpD* itself, no mutant is yet known which has lost TlpD function. *In vivo, H. pylori* tactic abilities are involved in modulating or avoiding the inflammatory host response against *H. pylori* which generates oxidative stress^11^,^13^,^19^,^64^. Oxidative stress and iron depletion both can act on the same cellular targets by affecting iron-sulfur cluster integrity and thereby the activity of essential metabolic dehydratase enzymes by damaging their active centres^44^,^45^. Among the most important iron-sulfur proteins in *H. pylori* are aconitase, fumarate reductase, hydrogenase, PFOR and the NifSU proteins themselves^46^,^65^. *H. pylori nifU* was an essential gene *in vitro*^46^,^66^ and hydrogenase was required for survival in a mouse model^67^. The modulatory effect of NifSU on TlpD behavioural activity corresponds to a major role of oxidative or iron stress with regard to TlpD functionality and requirement *in vivo*, as previously reported by us^13^ and others^12^. This mutuality implies a connection of TlpD function to iron-sulfur protein homeostasis, which, however, could not yet be pinpointed to any direct protein interaction in the present study. Future studies will have to clarify if interactions with other non-chemotactic proteins, possibly containing iron-sulfur clusters, are required for the sensing mechanism of TlpD.

### TlpD contributes to gene regulation in *H. pylori* by influencing multiple transcripts

An involvement of dedicated chemotaxis receptors in bacterial gene regulation has rarely been reported, and a role for TlpD in gene regulation has not been determined either. Direct or indirect functions of transducer-like proteins in gene regulation are conceivable, especially when the overall number of regulators is low, as in *H. pylori*. Prüss *et al*.^68^ described an effect of the *E. coli* aerotaxis sensor Aer on transcript amounts which may be linked to an important role in maintaining intrabacterial energy levels. On the basis of the assumption that gene regulation events can be helpful in deducing the TlpD sensing mechanism or protein interactions relevant to understanding its function, or finding causes of functional deficiencies of the *tlpD* mutant, we have tested how the transcriptome is affected in *H. pylori tlpD* mutants. In addition, the possibility of a regulatory compensation for the loss of energy taxis in *tlpD* mutants needed to be explored. In the *tlpD* mutant in comparison with the wild type strain, numerous differentially expressed transcripts in diverse functional categories were identified. The effects on transcript amounts were moderate rather than drastic which suggested an indirect role of TlpD. This raised the question of how the genes might be differentially regulated in the absence of TlpD. *nifU* and *katA* transcripts were positively affected by the loss of TlpD. Both *nifU* and *katA* were previously found positively regulated by the HP1021 orphan response regulator, whose sensory input is unknown^69^. Some of the changed transcripts in the *tlpD* mutant also appeared to overlap with the Fur or other metal-dependent regulons^70^,^71^. *nifSU* transcripts were earlier reported to be upregulated under conditions of oxidative stress, excess iron and by the ferric uptake regulator Fur^70^. Only a fraction of the transcript changes in the *tlpD* mutant can be linked to the canonical regulators HP1021 or Fur, which calls for other possible explanations. One way how TlpD might be linked to regulation is via its identified interaction with AcnB. As a basis, previous studies have indeed demonstrated that aconitase B in other bacteria (*E. coli*)^72^,^73^, mammals^74^, and recently also in *H. pylori*^49^, performs a central “moonlighting” role as a posttranscriptional gene regulator, in particular under conditions of iron depletion and oxidative stress. This moonlighting function was inversely correlated with AcnB catalytic activity, which is dependent on the integrity of its iron-sulfur complexes and determines its catalytic or regulatory protein conformation^72^. Austin *et al*.^49^ noted that the iron chelator 2,2-DP that we also observed to impact on TlpD sensing, can inactivate he catalytic activity *H. pylori* aconitase and enhance the regulatory function of aconitase. Some transcripts that we found higher expressed in *tlpD* mutants in microarrays or real-time PCR overlapped with results of a recent proteome analysis of an *acnB* mutant^75^, among those *ureA* (functional urease subunit gene), *tpx* (thiol peroxidase gene), *ahpC* (alkylhydroperoxidase subunit gene), *porA*, (pyruvate-ferredoxin oxidoreductase subunit gene A), *trx2* (thioredoxin 2 gene), *trxR2* (thioredoxin reductase gene). Thus it is plausible that the regulatory potential of AcnB in *H. pylori* might be harnessed by the TlpD interaction, which needs to be clarified. In addition, an intriguing finding of the latter study with regard to our results was that CheAY2, SecA and IleS, which we recovered in the TlpD pull-down, were all enriched in membrane fractions of an *acnB* mutant^75^. Thus, the finding of TlpD-dependent transcript changes also underlines a second hypothesis regarding the regulatory effect in the *tlpD* mutant and functions of TlpD in the context of confirmed protein-protein interactions: namely, that binding to TlpD might influence the catalytic status and regulatory activity of interacting proteins, for instance of AcnB (Fig. 7). In tune with this hypothesis, a slight reduction in AcnB catalytic activity was noted in *H. pylori* lysate of a TlpD overexpressing strain in contrast to the parent (own unpublished data).

Thus, in summary, a compensatory gene regulation in *tlpD* mutants was identified, possibly connected to AcnB-mediated posttranscriptional regulation, which strengthens a link of TlpD to metabolism, oxidative stress and iron provisioning.

## Conclusions

With TlpD and the acid sensor TlpB, two of four *H. pylori* behavioural receptors may observe a function possibly associated with intrabacterial energy status, indicating a prominent role of energy taxis for this bacterium in its challenging environment. Since major oxidative stress by the host defence and metal stress by varying metal ion availability *in vivo* are assumed to play a crucial role in the stomach niche of *H. pylori*, energy-associated behavioural functions are anticipated to exert a major influence on the short-term environmental flexibility of the organism *in vivo*. TlpD interacted with several other bacterial cytoplasmic proteins in addition to its expected interaction with the CheAY2 signalling complex. At present, combined data on TlpD localisation and behavioural activity in the tested mutants do not provide conclusive evidence as to where in the cell TlpD is active. Fine *in situ* localisation of CheAY2 and other interaction partners under various conditions will be required to interpret TlpD localisation in the context of TlpD function. A current model of TlpD interactions and metabolic crosstalk is presented in Fig. 7: confirmed TlpD interactors aconitase B and catalase both play an important role in metabolic functions and regulation or protect against iron and oxidative stress which threaten the bacterial central metabolism and respiratory chain (Fig. 7). Although we could not link any of the interactions, including KatA and AcnB, directly to the TlpD sensing and signalling mechanisms so far, TlpD affected multiple transcripts, possibly in part via AcnB-dependent regulation (Fig. 7). By fine-tuning posttranscriptional gene regulation, TlpD might act in a flexible manner on cellular functions which feed back into behaviour via expression changes in metabolism, redox functions and sensory proteins. Thereby, TlpD may provide a short to medium-term global adaptation process to metabolic threats, in case TlpD energy taxis functions are not sufficient to quickly evade these threats. In support of this, functional data demonstrated that sensing and the repellent mechanism initiated by the TlpD sensor are strongly connected to cellular functions depending on iron and damaged by oxidative stress. TlpD sensing is probably connected to at least one of the iron-sulfur cluster proteins in the central metabolism^45^,^46^,^65^ (Fig. 7), which can act as sensors for redox and iron^76^ and are intimately linked to the energetic status of the cell. Conditional depletion of specific essential iron-sulfur proteins and the structural investigation of TlpD will permit to clarify remaining questions concerning protein-protein interactions or functional implications of these proteins for energy-dependent behaviour.

## Methods

See Supplementary Methods for details about DNA and protein methods, protein-based pull-down-assays, construction of *H. pylori* allelic exchange mutants (Table 2, Supplementary Table S3), RNA preparation from *H. pylori* and cDNA synthesis, immunofluorescence labelling and microscopy^26^,^77^, and statistics.

### Mass spectrometry after TlpD pull-down

For tandem mass spectrometry, peptides recovered after TlpD-Hisx6 based pull-down and trypsin digest^78^ were dissolved in 2% ACN, 0.1% formic acid, and reverse phase chromatography using acetonitrile as an eluent was performed on a nanoACQUITY UPLC system (Waters) equipped with an analytical column (Waters, BEH130C18, 100 μm × 100 mm, 1.7 μm particle size), coupled online to an ESI Q-TOF (Q-TOF MS/MS; Ultima, Waters, Milford, MA, USA). Spectra were recorded in positive reflection mode, and peptides were automatically subjected to fragmentation (MS/MS). Protein and peptide identification were performed using the program ProteinLynx™ Global Server (Version 2.1, Waters) and the MASCOT search engine (Matrix Science). A more detailed description of peptide identification parameters, database, mass spectrometry settings and scoring is provided in the Supplementary Methods.

### Bacterial strains and growth conditions

*Helicobacter pylori* strains N6^79^ and HP87P7^13^ and their isogenic mutants are listed in Table 2. For details of growth conditions see Supplementary Methods.

### Purification of *H. pylori* TlpD-V5, AcnB-V5, Hisx6-TlpD and catalase using affinity chromatography

We purified recombinant Hisx6-TlpD (N-terminally fused Hisx6 Tag) from *E. coli* BL21(DE3) upon expression from a pET28a based plasmid (pCJ1341). TlpD-V5 and AcnB-V5 were affinity-purified from cleared lysates of *H. pylori* expressing TlpD-V5 or AcnB-V5 from plasmids. Catalase was purified after recombinant expression in *E. coli* as previously described^80^. For details of purification procedures, see Supplementary Methods.

### Testing of direct interactions of TlpD and interaction partners using biolayer interferometry

To test for direct protein-protein interaction, biolayer interferometry (BLI) was performed on the Octet RED96 System (ForteBio, Menlo Park, USA). BLI is an optical biosensing method that yields kinetic and affinity information in a manner similar to surface plasmon resonance (SPR)^42^. Purified recombinant *H. pylori* catalase (KatA)^41^,^80^ and V5-purified native *H. pylori* aconitase (AcnB-V5) were used as interaction partners and analytes for sensor-coupled Hisx6-TlpD (ligand). Alternatively, recombinant KatA (ligand) was tested with native TlpD-V5 (analyte) purified from *H. pylori*. Analyses of the binding curves were performed using the Octet Data Analysis Software 6.4.. For a detailed description, see Supplementary Methods.

### Single cell tracking and analysis of steady-state behaviour of *H. pylori*

The behaviour of *H. pylori* N6 and its derivatives in liquid medium was determined using established cell tracking protocols^26^. Briefly, freshly grown bacteria (grown about 20 h on blood agar plates) were resuspended in pre-warmed defined media at an OD_600_ of 0.03. Bacteria were visualised using a live cell microscope (Olympus IX-80 inverted Cell-R microscope) equipped with a climate chamber^26^. Before recording movies, bacteria were pre-incubated and equilibrated at 37 °C and ambient air enriched with 5% CO_2_ for 15 min. Thus, we determined for each condition a steady-state behaviour under equilibrated conditions. We did not observe an overt change of behaviour within the first 15 min of equilibration before the measurements were started. One might suspect that adaptation can occur during these 15 min. However, behavioural adaptation by sensor methylation is not known to occur in *H. pylori*, and *cheB*/*cheR* genes for methylating and demethylating Tlp sensors are not present in the *H. pylori* genome^16^. For quantification of bacterial traces, 3 to 4 movies (250 frames, 19 frames s^−1^), were recorded for each bacterial strain, and the swimming patterns of at least 50 single cells per strain (see figure legends) were analysed using the Cell-R system as described before^13^,^26^. We counted each single stop, and each stop in conjunction with a direction change, as one stop. In addition, we counted as one stop a full reversal of the bacteria (including one back–up movement, and one immediately following forward-run), if there was no direction change involved. Bacteria on the bottom of the field of view, which are bacteria in close contact with the solid support and could be sticking to the surface, were excluded from the counts and analyses. For the motility analysis of the strain N6 (pHel2::*nifSU*), we also tested an empty pHel2 plasmid as a control, which showed no behavioural difference in comparison to the parental N6 strain (data not shown).

### Testing growth and behaviour of *H. pylori* under iron-depleted conditions

*H. pylori* N6 wild type and *tlpD* mutant were tested for growth defects and behaviour in agar diffusion assays and steady-state tracking assays under iron-limited conditions by adding the iron-chelator 2,2-dipyridyl (Sigma-Aldrich, St. Louis, USA). For agar diffusion assays, bacteria grown on blood agar plates for about 20 h were resuspended in BHI medium to an OD_600_ of 0.07. 100 μl of the bacterial suspension were plated on Blood Agar Base No. 2 plates (Oxoid) supplemented with 5% horse serum. 10 μl of 10 mM, 20 mM and 40 mM 2,2-dipyridyl (dissolved in ethanol) as iron chelator or 10 μl of appropriate dilutions of ethanol in PBS (negative control) were applied to the plates on paper discs (diameter 5 mm). The diameter of the growth inhibition zone was measured upon incubation of bacteria at standard conditions for 48 to 72 h. Tracking assays under iron-limited conditions were performed as described above. After equilibrating the bacteria in liquid medium (see above), 0.5 mM or 5 mM 2,2-dipyridyl was added to the medium or the bacteria were left untreated. Movies were recorded 15 min and 30 min after the addition of 2,2-dipyridyl and steady-state behaviour was evaluated as described above. Steady-state assays with paraquat (10 μM, 100 μM) were carried out according to the same procedure.

### Transcript analysis using whole genome microarray

Comparative analyses of transcript levels in N6 and HP87 P7 and their respective *tlpD* mutants were performed using whole genome DNA microarrays as previously described^81^. Microarrays were generated and quality-controlled as previously described^81^,^82^. The custom-made whole-genome microarray consists of 1,649 gene-specific PCR products generated with specific primer pairs derived from the genome sequences of *H. pylori* strains 26695^16^ and J99^15^, which were all spotted in duplicates. Microarray hybridisation, raw data generation, and data analysis including normalisation and quantitation were performed as previously reported^81^,^83^. Raw data of the competitive hybridisations were analysed using ImaGene v5.0 (BioDiscovery, USA) and MAVI v2.5 (MWG Biotech AG, Ebersberg, Germany) using previously described settings^83^. Four microarray slides in total were evaluated in dual-colour mode for strain N6, and dyes were swapped between the parental strain and *tlpD* mutant in one of the four experiments. Finally, the mean values of four separate arrays, including three biological experiments and one dye swap (each array comprising in addition two technical replicates for each gene), of *H. pylori* N6 and its isogenic *tlpD* mutant were summarised and evaluated jointly for the final dataset (Supplementary Tables S1 and S2). Significance Analysis of Microarray (SAM^84^, Version 5.0) analysis was performed for the dataset with the following parameters: Data Type: One class; Arrays centered: true; delta: 0.32022; number of permutations: 100; input percentile for exchangeability factor s0: automatic; number of neighbours for KNN: 10; seed for random number generator: 1234567. These settings calculate a number of significantly regulated transcripts of 81, a probability of correct assignments of 95% and a false discovery rate (FDR) of 5.3%. Statistically significant changes are indicated for single transcripts in Supplementary Tables S1 and S2. Stringent criteria for the evaluation of regulated transcripts for the final dataset were set as lower and upper limits for the transcript ratios of 0.5- and 2-fold changed, respectively. Changes in transcript levels of selected regulated transcripts in microarray results of two *H. pylori* strains were additionally verified using semi-quantitative and quantitative RT PCR (Supplementary Fig. S4). Microarray data shown in the manuscript have been deposited in the GEO omnibus database (http://www.ncbi.nlm.nih.gov/geo/) under the accession number GSE79364.

### Quantitation of fluorescence intensities for single bacterial cells and statistics

Subcellular fluorescence distribution in *H. pylori* (visualised with fluorescently labelled anti-V5 by α-V5 antibody) was determined using ImageJ densitometry^85^of TIFF images, which were obtained using the same exposure and lighting settings for each strain. Measurements were then used to generate intensity histograms of TlpD for each cell along the longitudinal axis of at least 15 or 30 separate bacterial cells per strain and evaluated statistically. Details of quantitation methodology and statistics are described in the Supplementary Methods.

## Additional Information

**How to cite this article**: Behrens, W. *et al*. Localisation and protein-protein interactions of the *Helicobacter pylori* taxis sensor TlpD and their connection to metabolic functions. *Sci. Rep.* **6**, 23582; doi: 10.1038/srep23582 (2016).

## Supplementary Material

Supplementary Information

## Figures and Tables

**Table 1:** *H. pylori* proteins of which peptides were identified by mass spectrometry in two independent pull-down assays of TlpD-Hisx6 from *H. pylori tlpD* (TlpD-Hisx6) cleared cell lysates.

Protein	Number of identified peptides^a^
Histidine kinase (CheAY2)	2;1 (band 1)
peptides:	
Expt. 1: IYGDVEER; MDGYTFASEVR	
Expt. 2: ISQDEIYTVDGK	
Aconitate hydratase (AcnB)	5;8 (band 1)
peptides:	
Expt. 1: GVPVAYVGDVVGTGSSR; IPLIIGR;	
VTTVGSQDTTGAMTR; ILEIEGLPDIK;	
MEQAFELSDASAER	
Expt. 2: AEFLAQLSQK; DIPFVPNKR; IPLIIGR;	
VTTVGSQDTTGAMTR^ox^;MEQAFELSDASAER^ox^; LWVVPPSK	
Isoleucyl-tRNA synthetase (IleS)	3;2 (band 1)
peptides:	
Expt. 1: TSLENPTLFR; SAFNEELDR; LSETPAFTLFK	
Expt. 2: TSLENPTLFR; VEFVPSSGK	
Catalase (KatA)	1;1 (bands 2, 3)
peptides:	
Expt. 1: SLPADEKER	
Expt. 2: FSTVAGER	
Protein translocase (SecA)	1;1 (band 1)
peptides:	
Expt. 1: TPLIISGPVDR	
Expt. 2: TPLIISGPVDR	
DNA-dependent RNA polymerase (RpoBC)	-;7 (band 3)
peptides:	
Expt. 2: DSYDSFLYSK; VLSAYEEEK; KPETINYR;	
FAVSDVNELYR; VLDQGNIIATSAGR; NASILVVEPK;	
VSELFEAR	
Methyl-accepting protein TlpD (HylB)^b^	38;21 (bands 1, 2, 3)

Distinct protein bands of corresponding mass were excised for both experiments. Bands are indicated with three black arrows in Fig. 1, from top (1) to bottom (3). Only proteins which were detected in both replicate experiments, or which were identified with high score (>8; ProteinLynx) by more than 5 peptides in one single experiment, are listed. Only identification scores for proteins of equal or above 7 (ProteinLynx) were considered. The names of the identified proteins in the table correspond to the database designations. aExperiment 1; Experiment 2: bands 1,2, 3 designate bands that were excised from SDS gels after TlpD pull-down for mass spectrometry. For details of mass spectrometry analyses see Supplementary Methods.

^b^pull-down target (single peptides not listed).

^ox^respective peptide was identified twice - as an unmodified and as a methyl-oxidated variant.

**Table 2:** *Helicobacter pylori* strains and plasmids used in this study.

Strain or plasmid	Relevant characteristics	Source or reference
*H. pylori* strains and short designations	Strain characteristics and full designations	
N6	parental strain; human patient isolate	79
N6 *tlpD*	*H. pylori* N6 *tlpD::aphA3*	26
HP87P7	wild type, gerbil-adapted	13
HP87P7 *tlpD*	*H. pylori* HP87 P7 *tlpD::aphA3*	13
N6 *tlpD* (TlpD-Hisx6)	*H. pylori* N6 *tlpD::aphA3* (pHel2::*tlpD-hisx6*)	this study
N6 *tlpD* (pHel2::*tlpD-V5*)	*H. pylori* N6 *tlpD::aphA3* (pHel2::*tlpD-V5*)	26
N6 *rdxA::tlpD-V5*	*H. pylori* N6 *rdxA::tlpD-V5* Cm^R^ (chromosomal complementation)	this study
N6 *tlpD rdxA::tlpD-V5*	*H. pylori* N6 *tlpD::aphA3 rdxA::tlpD-V5* Cm^R^	this study
N6 *cheAY2 rdxA::tlpD-V5*	*H. pylori* N6 *cheAY2::aphA3 rdxA::tlpD-V5* Cm^R^	this study
N6 *tlpABC rdxA::tlpD-V5*	*H. pylori* N6 *tlpA::aphA3 tlpBC* (unmarked partial deletions) *rdxA::tlpD-V5* Cm^R^	this study
N6 *katA rdxA::tlpD-V5*	*H. pylori* N6 *katA::aphA3 rdxA::tlpD-V5* Cm^R^	this study
N6 *acnB rdxA::tlpD-V5*	*H. pylori* N6 *acnB::aphA3 rdxA::tlpD-V5* Cm^R^	this study
N6 *acxC rdxA::tlpD-V5*	*H. pylori* N6 *acxC::aphA3 rdxA::tlpD-V5* Cm^R^	this study
N6 (AcnB-V5)	*H. pylori* N6 (pHel2*::acnB-V5*) Cm^R^	this study
N6 *acnB*	*H. pylori* N6 *acnB::aphA3*	this study
N6 *katA*	*H. pylori* N6 *katA::aphA3*	this study
N6 *acxC*	*H. pylori* N6 *acxC::aphA3*	this study
N6 *cheAY2*	*H. pylori* N6 *cheAY2::aphA3*	26
N6 (NifSU^+^)	*H. pylori* N6 (pHel2::*nifSU*)	this study
**Plasmids**	**Plasmid characteristics**	
pUC18	Amp^R^, Rep_Ec_; high-copy-number vector	86
pUT18	Amp^R^; Rep_Ec_; pBR322 derivative; cloning vector	87
pILL570	Spec^R^; Rep_Ec_; cloning vector	88
pEF6-V5	Amp^R^, Blast^R^; Rep_Ec_; source of V5-tag sequence	Invitrogen Life Technologies, Darmstadt, Germany
pET28a	Km^R^; Rep_Ec_; high-copy-number expression vector, includes hexa-histidine tag sequence	Novagen
pHel2	Cm^R^; Rep_Ec_, Rep_Hp_; multi-copy-number vector; *H. pylori* expression plasmid	89
pKSF	Km^R^; Rep_Ec_; source of the *kan-sacB* construct	90
pCJ522	Cm^R^; *tlpD-V5* fusion gene including the potential *tlpD* promoter region in pHel2 shuttle vector	26
pCJ545	Cm^R^; *tlpD*-*hisx6* fusion gene including the potential *tlpD* promoter region in pHel2 shuttle vector	this study
pCJ1341	Km^R^; pET28a derivative; *tlpD* fused N terminally with Hisx6	this study
pCJ542	Amp^R^, Cm^R^; pUC18 derivative; *tlpD-V5* expression construct and *CAT* cassette inserted in *rdxA* flanking regions (for chromosomal complementation)	13
pCJ1306	Amp^R^, Km^R^; pUT18 derivative; *acnB* disrupted by *aphA3* cassette	this study
pCJ1308	Amp^R^, Km^R^; pUT18 derivative; *katA* disrupted by *aphA3* cassette	this study
pCJ537	Amp^R^, Km^R^; pUC18 derivative; *acxC* disrupted by *aphA3* cassette	this study
pCJ513	Spec^R^, Km^R^; pILL570 derivative; *tlpB* disrupted by *kan-sacB* construct (kanamycin resistant, sucrose sensitive)	this study
pCJ514	Spec^R^; pILL570 derivative; *tlpB* partially deleted (unmarked)	this study
pCJ1396	Amp^R^, Km^R^; pUC18 derivative; *tlpA* disrupted by *aphA3* cassette	this study
pCJ1314	Cm^R^; *acnB-V5* construct and potential *acnB* promoter region in pHel2 shuttle vector	this study
pCJ1350	Cm^R^; *nifSU* gene cluster including the potential promoter region in pHel2 shuttle vector	this study

Amp^R^ ampicillin resistance, Km^R^ kanamycin resistance, Cm^R^ chloramphenicol resistance, Blast^R^ blasticidin resistance, Spec^R^ spectinomycin resistance, Rep_Ec_ replication in *E. coli*, Rep_Hp_ replication in *H. pylori*.

**Table 3:** Distribution of TlpD-V5 between cell poles and cell body in *H. pylori* strains lacking potential interaction partners.

Genotype	Mean fraction of intensity at poles^a^/cell body^b^ [%] ± SD^c^	Significance (p value)^d^	Maximum amplitude as fraction of maximum absolute intensity
N6 *rdxA::tlpD-V5*	50.7/49.3 ± 21.7	–	83.5%
N6 *cheAY2 rdxA::tlpD-V5*	20.7/79.3 ± 7.1	2.2*10^–5^	not determined
N6 *tlpABC rdxA::tlpD-V5*	44.0/56.0 ± 17.2	ns	82.7%
N6 *katA rdxA::tlpD-V5*	27.6/72.4 ± 7.3	5.7*10^–4^	56.9%
N6 *acnB rdxA::tlpD-V5*	53.0/47.0 ± 9.7	ns	94.4%
N6 *acxC rdxA::tlpD-V5*	59.8/40.2 ± 12.2	ns	95.6%

^a^polar regions were defined uniformly as six pixel lengths of highest fluorescence intensity from both poles inward.

^b^cell body was defined as the rest of pixels along the longitudinal axis of each bacterium, after subtracting polar regions defined in ^a^.

^c^mean and standard deviation derived from 15 bacteria.

^d^Student’s *t* test for significance of difference (p value) between N6 *rdxA::tlpD-V5* and each of the mutants.

SD: standard deviation; ns: not significant.

**Table 4:** Growth inhibition of *H. pylori* N6 and HP87 P7 wild type, *tlpD* mutant and *tlpD* complemented strains in agar diffusion assays with different concentrations of the iron chelator 2,2-dipyridyl (DP).

Diameter of zone with growth inhibition [mm]^a^^,^^b^				
Strain	10 mM 2,2-DP	Significance (p value)	20 mM 2,2-DP	Significance (p value)
N6 wild type	9.6 ± 0.5		15.8 ± 1.0	
N6 *tlpD*	11.9 ± 0.9	0.017	17.8 ± 0.6	0.015
N6 *tlpD rdxA::tlpD-V5*	10.0 ± 0.0	0.205 (ns)	16.9 ± 0.3	0.118 (ns)
HP87P7 wild type	8.2 ± 0.2		16.1 ± 0.4	
HP87P7 *tlpD*	9.9 ± 0.7	0.029	18.3 ± 1.0	0.010
HP87P7 *tlpD rdxA::tlpD-V5*	10.1 ± 1.3	0.138 (ns)	17.7 ± 1.5	0.094 (ns)

^a^mean diameter of growth inhibition combined from duplicate experiments and standard deviation.

^b^p values for significance of difference calculated by Student’s *t* test in comparison to respective wild type.

ns: not significant.
