# Supplementary material for: Localisation and protein-protein interactions of the Helicobacter pylori taxis sensor TlpD and their connection to metabolic functions
Source: Sci Rep. 2016 Apr 5;6:23582. doi: 10.1038/srep23582 (PMC4820699; doi:10.1038/srep23582)
Supplement: Supplementary Information [file srep23582-s1.pdf]

SUPPLEMENTARY INFORMATION

Localisation and protein-protein interactions of the *Helicobacter pylori* taxis sensor TlpD and their connection to metabolic functions

Wiebke Behrens[1], Tobias Schweinitzer[1§], Jonathan L. McMurry[2], Peter C. Loewen[3], Falk F.R. Buettner[4], Sarah Menz[1], Christine Josenhans[1*]

[1]Institute of Medical Microbiology and Hospital Epidemiology, Hannover Medical School, Hannover, Germany
[2]Department of Molecular & Cellular Biology, Kennesaw State University, Kennesaw, GA, USA
[3]Department of Microbiology, University of Manitoba, Winnipeg, Canada
[4]Institute for Cellular Chemistry, Hannover Medical School, Hannover, Germany

Supplementary Methods

Growth conditions.

Bacteria were cultured on blood agar plates (Blood Agar Base No. 2, Oxoid, Wesel, Germany) supplemented with 10% horse blood (Oxoid) or in brain heart infusion broth (BHI, Oxoid) supplemented with 2.5 g l^{-1} yeast extract (Oxoid) and 10% horse serum (Biochrom, Berlin, Germany). Plates and liquid media were supplemented with antibiotics vancomycin (10 mg l^{-1}), polymyxin B (3.2 mg l^{-1}), trimethoprim (5 mg l^{-1}), and amphotericin B (4 mg l^{-1}); other selective antibiotics kanamycin (20 mg l^{-1}) and chloramphenicol (10 mg l^{-1}) were added when required. Microaerobic growth conditions were set up using Anaerocult C catalyst packs in air-tight jars (Merck, Darmstadt, Germany). Liquid cultures were usually started with an OD_{600} of 0.07 from overnight plate-grown bacteria as described in[1].

DNA methods.

DNA methods, such as plasmid and genomic DNA extraction from *H. pylori* and *E. coli* cells, PCR, and cloning were carried out following standard methods[2]. Plasmids were generated and propagated in *E. coli* DH5α[3], MC1061[4] or XL1-Blue (Agilent Technologies, Waldbronn, Germany). *E. coli* cells were grown on LB agar plates or in LB broth supplemented with appropriate selective antibiotics, kanamycin (20 mg l^{-1}), chloramphenicol (10 mg l^{-1}) or ampicillin (200 mg l^{-1}).

Construction of *H. pylori* allelic exchange mutants.

tlpD (HP0599), and *cheAY2* (HP0392) mutants were generated by insertion of an *aphA3* cassette (conferring kanamycin resistance) using allelic exchange mutagenesis in different *H. pylori* strains[5]. To construct a triple (*tlpABC*) transducer knockout strain, an unmarked partial deletion of *tlpC* was generated as described before[5] by the *km-sacB* counterselection procedure[6] and was carried out for *tlpB* (HP0103) analogously. Briefly, the *tlpB* locus was amplified and inserted into pILL570. The plasmid was inverse-amplified from the mid region of *tlpB* and ligated with the *kan-sacB* construct which was amplified from pKSF[6] (pCJ513). A second plasmid with a *tlpB* construct missing about 230 bp in the mid region of the gene was generated by religating an inverse-amplified fragment of pCJ513 (named pCJ514). After stepwise

generation of the unmarked *tlpBC* double mutant using the described procedure[5], *tlpA* (HP0099) was inactivated by insertion of the *aphA3* cassette as described before[5]. Isogenic allelic exchange insertion mutants in *katA* (HP0875), *acxC* (HP0697), *acnB* (HP0779) in *H. pylori* (strain N6) were created by allelic exchange mutagenesis in an analogous manner. Briefly, the gene sequences were amplified from genomic DNA using oligonucleotides adding restriction enzyme sites to the gene sequence (oligonucleotides are listed in Supplementary Table S3). The gene portions were cloned into the respective restriction sites of pUC18 or pUT18. Using either natural restriction sites or inverse amplification, the *aphA3* cassette was introduced into the central portion of each gene[7]. The resulting plasmid constructs pCJ1306, pCJ1308, and pCJ537 were used in the transformation of naturally competent *H. pylori*. Clones integrating the antibiotic resistance cassette into their respective chromosomal loci were selected on selective antibiotic plates. Correct introduction of the cassette was confirmed with PCR using at least two different primer combinations and excluding single cross-over recombination events.

Complementation of isogenic *tlpD* mutants with TlpD was accomplished either in *cis* or *trans*. The shuttle vector system of pHel2 (Cm^R)[8] was used to express TlpD from a plasmid. We have previously reported a TlpD-V5 fusion construct in the shuttle plasmid pHel2 (pCJ522)[5]. *Cis* complementation with a *tlpD-V5* gene fusion in the chromosomal *rdxA* locus was described before[1]. In addition, strains combining allelic exchange mutants in other loci (see above) with one copy of *tlpD-V5* in the *rdxA* locus were generated by recombination-mediated allelic exchange. These strains still express endogenous TlpD in addition to the V5-tagged version. To exclude effects of this specific expression approach for TlpD localisation and function, two control strains, with (N6 *rdxA::tlpD-V5*) and without (N6 *tlpD rdxA::tlpD-V5*) endogenous TlpD, were tested in parallel and showed similar results in all experiments (controls see Supplementary Fig. S1). To express TlpD as a C-terminal fusion with a hexa-histidine tag (Hisx6) in *H. pylori*, the complete pHel2 expression plasmid containing the TlpD-V5 construct (pCJ522) was reverse-amplified (pCJ522_His_fw/pCJ522_His-rev2), omitting the V5 tag sequence. Primer sequences were then designed to include the Hisx6 sequences, which, upon self-ligation of the PCR product, resulted in a circular plasmid carrying the potential *tlpD* promoter and an in-frame fusion of the complete *tlpD* gene, a short linker sequence as previously

used for expressing a C-terminal MCP fusion[9], and the Hisx6-tag (pCJ545). To enable self-ligation, the PCR product was incubated with T4 polynucleotide kinase (NEB, Bad Schwalbach, Germany).

To generate a Hisx6-TlpD expression plasmid for recombinant protein purification from *E. coli*, the complete *tlpD* gene (only leaving out the start codon) was amplified from strain 26695. The fragment was inserted into the pET28a expression vector using BamHI/NotI restriction sites, resulting in an N-terminal in-frame fusion of the hexa-histidine tag with *tlpD* (pCJ1341). For an *acnB-V5* expression construct for *H. pylori*, *acnB* and 160 nucleotides upstream of the start codon were amplified from *H. pylori* 26695. The V5 tag, its linker region and a pHel2 backbone were amplified from a previous construct pCJ607[5] in an inverse PCR, resulting in an opened plasmid. During both amplification processes, SpeI restrictions sites were added and allowed to generate a circular pHel2 vector with in-frame fusion of *acnB*, linker and C-terminal V5 tag (GKPIPNPLLGLDST)[9] (pCJ1314). Due to the cloning strategy, the linker sequence was slightly modified and elongated by one nucleotide triplet (linker coding for: GGLVSAAG) compared to previously used linkers[13]. To generate NifSU expression clones, the *nifSU* gene cluster and 300 bp of the upstream region were amplified from 26695 and cloned into pHel2 (pCJ1350). All bacterial strains and plasmids are listed in Table 2.

H. pylori clones that had taken up plasmids or incorporated DNA by recombination were selected on antibiotic plates. DNA of recombinants was validated as described above and the presence of proteins in knockout and expression clones, respectively, were verified by Western blots. The strains were further characterised for growth and motility in growth curves and live microscopy. The intact behavioural phenotype of the TlpD-V5 fusion in *H. pylori* was verified before[5]. We also confirmed that the functionality of TlpD-Hisx6 (in the N6 *tlpD* mutant) was maintained in comparison to non-tagged TlpD using single cell tracking analysis (data not shown). All mutants that were chosen for further analyses did not show major differences in growth or motility compared to the parental strain.

Protein methods.

Standard procedures were followed for the determination of proteins amounts and separation of proteins on SDS gels (see also Supplementary Methods below for details of antibodies used for protein detection).

Pull-down assay for protein-protein interaction and mass spectrometric analyses.

Cleared cell lysates of *H. pylori* N6 *tlpD::aphA3* (pHel2::*tlpD-hisx6*) and *H. pylori* N6 *tlpD::aphA3* (negative control) were prepared for a protein pull-down approach. For this purpose, plate-grown bacteria were resuspended in RIPA lysis buffer (100 mM NaCl, 25 mM Tris/HCl pH 7.5, 20 mM imidazole, 10% glycerol, 1% Nonidet P-40, Complete Mini protease inhibitor cocktail (Roche) and lysed by incubation on ice for 15 min and subsequent sonication. Insoluble cell debris was removed by centrifugation (10,000 x g, 20 min, 4°C). The cleared lysates (about 700 µg of total protein containing about 3.5 µg of TlpD-Hisx6) were mixed with TALON matrix (immobilised Co^{2+} ions, binding up to 5 µg of Hisx6-tagged protein per µl) (BD Biosciences, Erembodegem, Belgium), which was pretreated by washing three times with cold phosphate-buffered saline (PBS) (3,000 × g, 1 min, 4°C). Talon beads and bacterial lysates were co-incubated for 2 h at 4°C with gentle rotation. Affinity matrix and bound proteins were washed three times with RIPA buffer (see above) without Nonidet P-40 and imidazole, and two times with PBS (3,000 × g, 1 min, 4°C). Since we did not want to lose any of the native bead-bound material, we directly separated the final sediment of matrix and bound proteins on denaturing SDS gels after boiling in SDS sample buffer (including β-mercaptoethanol), and subsequently stained it with Coomassie blue. The pull-down assay was carried out in three independent replicates. The precipitated proteins were further analysed using mass spectrometry as follows. After visualising the captured proteins of N6 *tlpD* (TlpD-Hisx6) and N6 *tlpD* in SDS gels, protein bands that only occurred in N6 *tlpD* (TlpD-Hisx6) but not in N6 *tlpD* were cut out of the gels and subjected to mass spectrometry to identify potential TlpD-Hisx6 protein interaction partners. Before mass spectrometry, proteins were trypsin-digested over night[10]. For tandem mass spectrometry, peptides were dissolved in 2% ACN, 0.1% formic acid, and reverse phase chromatography using acetonitrile as an eluent was performed on a nanoACQUITY UPLC system (Waters)

equipped with an analytical column (Waters, BEH130C18, 100 µm × 100 mm, 1.7 µm particle size), coupled online to an ESI Q-TOF (Q-TOF MS/MS; Ultima, Waters, Milford, MA, USA). Spectra were recorded in positive reflection mode, and peptides were automatically subjected to fragmentation (MS/MS). Protein and peptide identification were performed using the program ProteinLynx™ Global Server (Version 2.1, Waters) and the MASCOT search engine (Matrix Science) using the NCBInr database (settings: type of search: Peptide Mass Fingerprint; enzyme: trypsin; fixed modifications: carbamidomethyl (C); variable modifications: oxidation (M); mass values: monoisotopic; protein mass: unrestricted; peptide mass tolerance: ± 0.2 Da; peptide charge state: 1+; maximum missed cleavages: 1; number of queries: 111). A probability-based Mowse Score was applied, where the protein score was -10*Log(P), and P was the probability that the observed match was a random event. Protein scores greater than 81 were considered significant ($p<0.05$). Since proteins from strain N6 that we used for the pull-down are not specifically included in the NCBInr database, we expected not to detect all possible peptides by theoretical mass, due to frequent amino acid differences between orthologous proteins of evolutionarily unrelated *H. pylori* strains. Two independent mass spectrometry analyses from two separate biological pull-down experiments were performed and analysed.

Purification of *H. pylori* TlpD-V5, AcnB-V5, Hisx6-TlpD and catalase using affinity chromatography.

We purified recombinant Hisx6-TlpD (N-terminally fused Hisx6 tag) from *E. coli* BL21(DE3) upon expression from a pET28a based plasmid (pCJ1341). Briefly, bacteria were resuspended in lysis buffer (50 mM Tris/HCl pH 7.5, 150 mM NaCl, 2 mM DTT, 5 mM $MgCl_2$) and lysed by sonication (5 x 2 min, 4°C). After separation into soluble and insoluble fractions (9,000 x g, 20 min, 4°C), Hisx6-TlpD was purified from the TlpD-enriched insoluble fraction using urea-containing solubilisation buffer (10 mM Tris/HCl pH 8.0, 100 mM NaH_2PO_4, 8 M urea) and a Protino Ni-IDA 2000 Packed Column (Macherey-Nagel, Düren, Germany). The column and proteins were washed three times (20 mM Tris/HCl pH 6.3, 100 mM NaH_2PO_4, 6 M urea) before the bound Hisx6-TlpD was eluted by decreasing the pH to 5.8 and finally to 4.5. Urea was removed by dialysing three times against 1 liter of lysis buffer (see above) in a

Slide-A-Lyzer cassette (Thermo Fisher Scientific, Waltham, USA), in the absence of DTT, due to the large volume needed for the dialysis. In later steps, 2 mM DTT was added again to the buffer. We also established a non-denaturing purification protocol for TlpD-Hisx6, which used larger culture volumes and the same buffer but without urea. Elution, in this case, was performed by adding imidazole (100 mM) to the elution buffer. However the TlpD obtained by non-denaturing purification was much less soluble and less pure, therefore we did not use it for the protein interaction analyses. Native TlpD-V5 and AcnB-V5 were purified from *H. pylori* N6 *tlpD* (pHel2::*tlpD-V5*) and *H. pylori* N6 (pHel2::*acnB-V5*) soluble fractions using the V5-tagged Protein Purification Kit (MBL International Corporation, Woburn, USA) according to the manufacturer's protocol. Briefly, soluble fractions were co-incubated with α-V5 tag beads on a mixing wheel for 1 h at 4°C. After several washing steps with the supplied washing buffer (all centrifugation steps were carried out at 4,000 x g for 10 s), V5-tagged proteins were eluted upon addition of a V5 elution peptide. The amount of purified proteins was determined in stained gels or Western Blots using α-V5 antibody (monoclonal mouse; Invitrogen Life Technologies, Darmstadt, Germany) or α-His antiserum (polyclonal rabbit; Rockland). The purity of the eluate was verified by SDS gels and Coomassie or silver staining. The purity of the proteins was estimated to be at least 95% pure or higher by silver staining. *H. pylori* catalase was recombinantly expressed and purified as previously described[11].

Testing of direct interactions of TlpD and interaction partners using biolayer interferometry.

To test for direct protein-protein interaction, biolayer interferometry (BLI) was performed on the Octet RED96 System (ForteBio, Menlo Park, USA). BLI is an optical biosensing method that yields kinetic and affinity information in a manner similar to surface plasmon resonance (SPR)[12]. Purified recombinant *H. pylori* catalase (KatA)[11] and V5-purified native *H. pylori* aconitase (AcnB-V5) were used as interaction partners and analytes for sensor-coupled Hisx6-TlpD (ligand). Coupled sensors were first dipped into ForteBio kinetics buffer (PBS, 0.1% BSA, 0.02% Tween20 and 0.05% sodium azide, pH 7.4) to establish a stable baseline, then into different concentrations of purified proteins (AcnB or KatA, respectively, diluted in kinetics buffer) and finally in pure kinetics buffer again to monitor dissociation. Both

association and dissociation processes were monitored over 20 min to 30 min. All measurements were carried out at 30°C with agitation. Baseline shifts as measured by a ligand-bound sensor dipped into buffer only in all phases were minimal and subtracted from responses to analytes. Analyses of the binding curves were performed using the Octet Data Analysis Software 6.4. The following fitting model (global fit) was used for both TlpD-AcnB and TlpD-KatA binding kinetics (two state parallel model (heterogeneous ligand)):

For the association phase:

$$y = \mathrm{Rmax}\,\frac{1 * (1 - e^{-(\mathrm{ka}*[\mathrm{Analyte}]+\mathrm{kd})x})}{1 + \frac{\mathrm{kd}}{\mathrm{ka} * [\mathrm{Analyte}]}}$$

For the dissociation phase: $y = y0 * e^{-kd(x-x0)}$, where

$$y0 = \mathrm{Rmax}\,\frac{1 * (1 - e^{-(\mathrm{ka}*[\mathrm{Analyte}]+\mathrm{kd})x0})}{1 + \frac{\mathrm{kd}}{\mathrm{ka} * [\mathrm{Analyte}]}}$$

The equation to fit a 2:1 binding interaction implemented in the Octet software uses a combination of two 1:1 curve fits, with an additional parameter to account for the percentage of binding contributed by each interaction.

To exclude non-specific effects of the set-up, the measurement was also carried out with a switch between ligand (bound to sensor) and analyte (added in buffer). For this purpose, Hisx6-free ligand (KatA) was coupled to the sensor surface (AR2G sensors) via free amine groups using the amine coupling kit according to the manufacturer's protocol, and TlpD-V5 was allowed to interact as analyte. Glutathione-S-transferase (GST), an unrelated bacterial protein which we purchased as a highly purified preparation (Sigma-Aldrich), was used as control analyte with sensor bound Hisx6-TlpD, and did not show any binding to TlpD (data not shown).

Immunofluorescence microscopy.

To analyse the bacterial subcellular localisation of TlpD-V5 in intact cells of N6 *rdxA::tlpD-V5* (reference) and derivative deletion mutants, immunofluorescence microscopy with immuno-labelled TlpD-V5 was carried out similarly to previous procedures[5,13]. A C-terminal TlpD-V5 fusion was expressed from the chromosomal *rdxA* locus. Bacteria, pre-grown on blood agar plates for about 20 h, were either immediately resuspended in fixing reagent (2% freshly prepared paraformaldehyde

in 100 mM potassium phosphate buffer, pH 7.0) or transferred to variable incubation conditions, such as different liquid media, BHI/yeast extract + 3% fetal calf serum or RPMI 1640 + 3% fetal calf serum for 20 min. Alternatively, media were supplemented with inhibitors myxothiazol (Sigma) or paraquat (Sigma) and incubated for another 10 or 30 min, respectively, as indicated in the results text and figure legends. All incubations were carried out at 37°C and ambient air enriched with 5% CO_2, before bacteria were directly diluted in fixing reagent. Bacteria in fixing reagent were adjusted to $2*10^8$ cells per 400 µl and attached to cover slips (pre-coated with 0.3% gelatin) for 3 h at room temperature. After washing with quenching buffer (0.1% glycine in PBS), bacteria were incubated in 0.1% Triton X-100 for 30 min at room temperature to increase permeability. Bacteria were washed with PBS and incubated with blocking reagent (1% bovine serum albumin, 1% fetal calf serum in PBS) for 30 min, followed by the first labelling step with α-V5 monoclonal antibody (mouse monoclonal; 1:1,000; Invitrogen), diluted in blocking reagent, for at least 16 h at 4°C. After several wash steps with 0.1% bovine serum albumin (BSA) in PBS, the specimens were incubated with a secondary goat α-mouse IgG antibody coupled to Alexa488 dye (1:5,000, Invitrogen) for 1 h. Subsequently, bacteria were subjected to additional wash steps with 0.1% BSA in PBS and PBS, and counterstained with FM4-64 SynaptoRed membrane stain (1:5,000, Biochrom, Berlin, Germany) in PBS for 30 min, followed by a final PBS wash. To preserve the stained bacteria, cover slips were placed in mounting medium (2.5% DABCO in Mowiol 4-88) on glass slides and dried overnight. Images were acquired with an Axio Imager.Z1m microscope equipped with an ApoTome at 63x magnification using the AxioCam MRm System and AxioVision Software (v4.8) (Carl Zeiss Microscopy GmbH, Jena, Germany). *H. pylori* N6 wild type was used as negative control for TlpD-V5 staining. A fixed exposure time specific for each dye was set to be able to compare different strains in some experiments.

RNA preparation from *H. pylori* and cDNA synthesis.

Bacteria were grown in liquid culture as indicated above to an OD_{600} of ~1.0 and harvested by centrifugation at 22,000 x g for 1 min at 4°C. The cell pellet was immediately frozen at -80°C until further use. Total RNA was prepared using a modified RNeasy spin column protocol (Qiagen, Hilden, Germany) as described

elsewhere[14]. The amount and purity of the isolated RNA was determined photometrically and on agarose gels. PCR was used to check for DNA contamination with the primer pair OLHPFlaA_4/OLHPFlaA_9[15]. To eliminate DNA contamination, DNase treatment with DNase I (Roche) and subsequent RNeasy column clean-up (Qiagen) were performed at least once.

In order to generate cDNA for microarray analyses, random hexamer primers (Invitrogen) were mixed with 15 to 25 µg of total RNA. After preincubation at 65°C for 10 min, and 10 min at room temperature, reverse transcription was carried out at 42°C for 2 h using the Superscript III system (Invitrogen). Fluorescence dyes Cy3 and Cy5 (0.5 mM, Amersham Pharmacia) were directly incorporated into cDNA during reverse transcription. The enzyme was inactivated and RNA was degraded by alkaline lysis. The reaction was cleared from enzymes, primers and nucleotides using the Qiagen PCR Purification Spin Kit (Qiagen). Equal amounts of differentially labelled cDNA of wild type and *tlpD* mutant were mixed, dried and stored at -20°C until further use in microarrays. Synthesis of cDNA for use in quantitative reverse transcriptase (qRT) PCR (see Supplementary Methods below) was carried out using 1 to 2 µg of total RNA according to the same protocol but without incorporating the fluorescent dyes.

Semiquantitative and quantitative real-time PCR.

For semi-quantitative RT PCR (sqRT-PCR), the standard PCR protocol was applied using equal amounts of parent and mutant cDNA in parallel samples. Gene-specific oligonucleotide pairs were used to amplify gene fragments; amplification cycle numbers were adjusted to each of these to stay below the saturation state of amplification. Amplification levels were analysed by agarose gel electrophoresis. For the quantitative comparison of transcript levels, qRT-PCR was performed. Adjusted amounts of cDNA were mixed with gene-specific primer pairs and SybrGreen (Qiagen) and analysed in a BioRad C1000/CFX96 combined real-time PCR system (BioRad, Hercules, USA). All samples were analysed as technical duplicates, and standard curves were generated from defined amounts of PCR product of the respective genes to allow quantitation. Values were normalised to *H. pylori* 16S rRNA gene control amplifications of all samples. Oligonucleotides used for PCR and qRT-PCR are available on request.

Crude fractionation of *H. pylori* and detection of proteins.

H. pylori cells, grown on plates for less than 24 h, were resuspended in 100 mM Tris/HCl (pH 7.4) supplemented with protease inhibitor cocktail (Complete mini, EDTA-free, Roche, Mannheim, Germany) and lysed in an ultrasonicator (Branson Sonic Power Company, Danbury, USA) for 30 s to 2 min at 4°C and power level 5 (80-100 W). Sonication steps were intermitted by cooling on ice and repeated up to five times. Equal amounts of 10 µg protein per sample were separated on 11.5% SDS polyacrylamide gels. After protein separation, proteins were either stained with Coomassie blue (Roth, Karlsruhe, Germany) or blotted on Whatman Protran BA85 (pore size 45 µm) nitrocellulose membrane (Schleicher & Schuell, Germany) using standard procedures. Immunolabelling of proteins on the blot membranes was performed using the following primary antisera and peroxidase-conjugated secondary antisera: α-TlpD (rabbit polyclonal; 1:10,000[1]), α-V5 (mouse monoclonal; 1:50,000; Invitrogen Life Technologies, Darmstadt, Germany), α-*E. coli* AcnB (rabbit polyclonal; 1:10,000; kindly provided by Jeffrey Green[16]), α-FlhA (polyclonal rabbit; 1:2,500;[17]); α-HopZ-II (polyclonal rabbit; 1:5,000;[18]); goat α-rabbit IgG (1:20,000; Jackson Immunoresearch Europe, Newmarket, UK); goat α-mouse IgG (1:20,000; Jackson Immunoresearch Europe, UK). Detection of *H. pylori* catalase in Western Blot analyses was achieved using conjugate solution from RIDASCREEN FemtoLab stool antigen test (R-Biopharm AG, Darmstadt, Germany; 1:40 diluted in 5 % skim milk, antibodies in conjugate are directly coupled to horseradish peroxidase). When indicated, bacterial whole cell lysates were fractionated into soluble (cytoplasmic enriched) and insoluble (membrane-enriched) crude fractions. In this case, crude lysates were prepared as described before[19], and the lysis of more than 95% of the bacteria, visible as ghosts or debris, was checked by light microscopy. Whole cell lysates were centrifuged at 100,000 x g and 4°C for 30 min to separate the fractions into pellets (insoluble fraction) and supernatants (soluble fraction). For all samples, equal amounts of 10 µg protein for both fractions were loaded on 11.5% SDS polyacrylamide gels. The fractions were controlled for specific protein enrichment by immunodetection of the membrane-associated FlhA, outer membrane protein HopZ, and cytoplasmic enzyme AcnB in Western Blot analyses. Densitometry of Western blot results was performed using ImageJ software (NIH, Bethesda, Maryland[20]). Anti-

H. pylori antiserum (polyclonal rabbit; Dako, Glostrup, Denmark) was used as a loading control for each sample and for normalisation of TlpD amounts in densitometry.

Statistics.

Differences in motile behaviour between various *H. pylori* strains in temporal assays were statistically evaluated using Student's *t* test (unpaired, two-sided). Levels of significance for numbers of cells with polarly localised TlpD clusters were calculated using Fisher's exact test (Fig. 3C). The statistical evaluation of altered distribution of TlpD in intact bacterial cells was performed as follows: ImageJ densitometry[21] of TIFF images, obtained using the same exposure and lighting settings for each strain, was used to generate intensity histograms of TlpD for each cell (visualised with labelled α-V5 antibody) along the longitudinal axis of at least 15 separate bacterial cells per strain. For each data point on the longitudinal axis, all pixels within the transversal axis were summarised and averaged. All subsequent calculations were performed in Microsoft Excel. All longitudinal data points of each bacterium were arranged on an x-axis of a fixed length set to 1 (100%), to be able to compare between bacteria of different lengths. For all single bacteria, pixel intensities were summarised separately for the polar regions (poles defined as the length of 6 pixels with the highest fluorescence intensities from both ends of the bacteria inwards) and the mid-cell body region (all residual pixels after subtraction of the polar regions) and calculated as the fraction of whole cell intensity (Table 3). Average and standard errors for each strain were calculated from the intensities of 15 bacteria, and the significance of the relative polar and cell body distributions of intensity between the different strains were determined using Student's *t* test (unpaired, two-sided). Additionally, an intensity profile (fitted trend line) as shown in Fig. 3B and Supplementary Fig. S2 was determined for each bacterium separately, using all data points on the x (longitudinal) axis of the intensity histogram. The highest available polynomial algorithm was used to fit the trend lines to the actual data points. Subsequently, a mean intensity profile for all bacteria of one strain was calculated by averaging the data points for all single intensity profiles, including standard deviations for each data point on the x-axis, depicted in Fig. 3B (see also Supplementary Fig. S2, for a comparison of single bacteria intensity histograms and

for average intensity profiles of the unfitted data). As an additional measure for differences in TlpD distribution between the polar and the cell body region, the maximum amplitude of intensity from the absolute maximum to the relative minimum within one cell was calculated (values shown in Table 3). The amplitude is indicated as a percentage of the absolute maximum value of each cell. For these calculations, the average intensity profile depicted in Supplementary Fig. S2 was used (Table 3). To quantitate differences in the fluorescence intensity of the cell body between different incubation conditions, intensities of all pixels of the bacterial cell excluding the poles were summed up and calculated as percent of the summarised pixel intensities of the whole cell including the poles (poles were defined in these experiments as a fixed length at each pole of six pixels with the highest fluorescence intensities). In each case, values of 30 bacteria per setting were averaged for cell body intensities, and the value of the reference strain or reference condition for each set of experiments was set to 100%. The other cell body intensities were then normalised and are given in percent of the respective reference strain or condition. Significance of differences was calculated by Student's *t* test (unpaired, two-sided).

Supplementary Materials

Supplementary Tables

Supplementary Table S1. Genes with increased transcript amounts in *H. pylori* N6 *tlpD* compared to N6 parental strain identified in competitive microarray analyses.

locus name	gene name	gene function	mean expression ratio[a] wt/*tlpD*	mean standard deviation
HP0014		hypothetical protein	0.49	0.21
*HP0073[#]	*ureA*	urease, alpha subunit	0.43	0.13
HP0084	*rplM*	ribosomal protein L13	0.48	0.13
HP0086	*mqo*	malate:quinone oxidoreductase	0.49	0.10
HP0116	*topA*	DNA topoisomerase I	0.46	0.06
HP0296	*rplU*	ribosomal protein L21	0.38	0.24
*HP0390[#]	*tpx*	adhesin / thiol peroxidase	0.47	0.08
*HP0546	*cagC*	*cag* pathogenicity island protein *cag25*	0.46	0.09
HP0596		tumor necrosis factor alpha-inducing protein	0.49	0.19
HP0599	*tlpD*	transducer-like protein (insertion mutant)	0.49	0.22
*HP1164	*trxR2 / fqrB*	thioredoxin reductase / flavodoxin quinone reductase	0.48	0.09
HP1187		hypothetical protein	0.41	0.28
*HP1192		secreted protein involved in flagellar motility	0.49	0.24
HP1304	*rplF*	ribosomal protein L6	0.50	0.31
HP1314	*rplV*	ribosomal protein L22	0.50	0.18
HP1316	*rplB*	ribosomal protein L2	0.50	0.24
HP1318	*rplD*	ribosomal protein L4	0.47	0.17
HP1561	*ceuE*	iron(III) ABC transporter, periplasmic iron-binding protein	0.50	0.20
HP1588[#]		conserved hypothetical protein	0.45	0.14
JHP0928		hypothetical protein	0.49	0.23

[a] Transcript levels from 4 independently performed microarrays of *H. pylori* N6 wild type (wt) and *tlpD* mutant including technical duplicates of each gene on each array were included to calculate the mean expression ratios (and standard deviation) of parental strain (wt) vs. *tlpD* mutant. Ratios equal or lower than 0.5 (wt/*tlpD*) are depicted. Using SAM settings of: delta 0.32022, False Discovery Rate 5.3%, q value 0.0 to 5.3% (as for data in Supplementary Table 2), none of the detected events was significant. # significantly changed protein levels in *H. pylori acnB* mutant[16]. * genes verified by qRT-PCR (see also Supplementary Fig. S6)

Supplementary Table S2. Genes with decreased transcript amounts in *H. pylori* N6 *tlpD* compared to N6 parental strain identified in competitive microarray analyses.

locus name	gene name	gene function	mean wt/*tlpD* expression ratio[a]	standard deviation
HP0012	*dnaG*	DNA primase	11.40	12.54
HP0017	*virB4*	DNA transfer protein	2.11	0.32
HP0030		hypothetical protein	2.06	2.84
**HP0038**	***comB1***	**hypothetical protein**	**2.52**	**2.02**
HP0040	*comB2*	hypothetical protein	2.30	1.64
**HP0052**		**hypothetical protein**	**2.59**	**1.02**
HP0059		hypothetical protein	4.09	2.96
**HP0081**		**hypothetical protein**	**2.87**	**1.84**
**HP0091**	***hsdR***	**type II restriction enzyme R protein**	**4.28**	**4.33**
**HP0093**	***futC***	**α-(1,2) fucosyltransferase**	**2.55**	**1.25**
**HP0261**		**hypothetical protein**	**2.29**	**1.51**
**HP0308**		**hypothetical protein**	**2.03**	**0.93**
HP0344		hypothetical protein	2.10	1.16
**HP0413**		**transposase-like protein**	**2.30**	**1.24**
**HP0414**		**IS200 insertion sequence from SARA17**	**2.01**	**1.59**
**HP0437**	***tnpA***	**IS605 transposase**	**2.28**	**0.40**
**HP0439**		**hypothetical protein**	**2.05**	**0.31**
**HP0441**		**VirB4 homolog**	**2.30**	**1.06**
HP0456		hypothetical protein	2.99	2.45
**HP0458**		**hypothetical protein**	**2.34**	**1.57**
**HP0475**	***modD***	**molybdenum ABC transporter, ATP-binding protein**	**2.71**	**1.13**
**HP0503**		**hypothetical protein**	**2.48**	**1.65**
**HP0505**		**hypothetical protein**	**2.87**	**1.59**
**HP0539**	***cagL***	**cag pathogenicity island protein *cag18***	**2.30**	**0.49**
**HP0676**		**methylated-DNA-protein-cysteine methyltransferase**	**2.71**	**1.52**
**HP0685**	***fliP***	**flagellar biosynthetic protein**	**2.01**	**0.76**
HP0878		hypothetical protein	2.76	1.65
**HP0880**		**hypothetical protein**	**2.05**	**1.26**
HP0885	*mviN*	virulence factor mviN protein	2.80	2.91
**HP0894**		**conserved hypothetical protein, TA system toxin**	**2.14**	**1.64**
**HP0897**		**hypothetical protein**	**2.40**	**0.34**
**HP0963**		**hypothetical protein**	**2.00**	**0.79**
HP0988	*tnpA*	IS605 transposase	2.04	2.17
HP0991		hypothetical protein	2.04	1.99
HP0992		hypothetical protein	3.67	2.66
HP0996		hypothetical protein	2.43	1.38
HP1074		hypothetical protein	2.49	1.54
HP1078		hypothetical protein	2.56	2.27
**HP1223**		**hypothetical protein**	**2.06**	**0.87**
**HP1224**	***hemD***	**uroporphyrinogen III cosynthase**	**2.12**	**1.28**
**HP1231**		**DNA polymerase III delta prime subunit**	**2.07**	**0.33**
**HP1252**	***oppA***	**oligopeptide ABC transporter substrate-binding protein**	**2.61**	**2.41**
HP1291		conserved hypothetical protein	2.80	1.88
HP1384		hypothetical protein	2.12	0.15

HP1420	*fliI*	flagellum-specific ATP synthase	2.73	3.12
JHP0925		hypothetical protein	2.02	1.97
JHP0926		hypothetical protein	2.07	0.69
JHP1462		hypothetical protein	31.19	41.68

[a] Expression data from 4 independently performed microarrays with *H. pylori* N6 wild type (wt) and *tlpD* mutant including technical duplicates of each gene on each array were included to calculate the mean expression ratios (and standard deviation) of parental strain vs. *tlpD* mutant. Ratios equal or higher than 2.0 (wt/*tlpD*) are depicted. Significantly altered transcript amounts (SAM) are highlighted in bold. SAM settings: delta 0.32022, False Discovery Rate 5.3%, q value 0.0 to 5.3% (as for Table S1).

Supplementary Table S3. Oligodesoxynucleotide primers for cloning.

Designation	Sequence[a]	Purpose
OL_pCJ522_His_fw	CATCACCATTGAGGATCCTCTAGAGTCGAC	construction of TlpD-Hisx6 fusion in pCJ545
OL_pCJ522_His-rev2	ATGATGGTGACCTGCTGCTGAAGATCCTCC	
OLHPTlpD_BamHI_F2	AAAGGATCCTTTGGGAATAAGCAGTTGCAAC	construction of Hisx6-TlpD fusion in pCJ1341
OHPTlpD_NotI_R1	AAAGCGGCCGCCACTTCATTGAATAAAATCATTCG	
OLHP_acnB_BamHI_F1	AAAGGATCCATGATGAAAGATTTTTTAGA	cloning of pCJ1306 for *acnB* mutants
OLHP_acnB_EcoRI_R1	AAAGAATTCGAGAGCCTGAAATTCTCCAT	
OLHP_acnB_inv3	TCCCCCGGGCACATACGCTACAGGAACG	
OLHP_acnB_inv4	TCCCCCGGGCCAATATCAAGCTGATTGATG	
OLHP_katA_XbaI_F1	AAATCTAGAGATGGTTAATAAAGATGTGAAAC	cloning of pCJ1308 for *katA* mutants
OLHP_katA_EcoRI_R2	AAAGAATTCAGCTTTTTCTTTTTTGTGTGG	
OLHP_katA_inv1	AAAAGATCTCCATGTCGTGGTTAGGCA	
OLHP_katA_inv2	AAAAGATCTGCAGAAGTGGAGCAAGTGG	
OLHP0697_1s	GAGAGATCTCCAGAATACGCTTACAAGG	cloning of pCJ537 for *acxC* mutants
OLHP0697_2s	GAGAGATCTGCTTTTCCTTGTTAGGTGG	
OLHP0697_3s	AGAGGATCCTGTTTGTGGTGCAAGATGC	
OLHP0697_4s	GAGGGATCCACACCTTCAGATACATTTGG	
OLHPAcnB_Spe_fw	GGACTAGTCATGTTCAAATAATCAATTTTACC	cloning of AcnB-V5 fusion in pCJ1314
OLHPAcnB_Spe_rv	GGACTAGTCCTCCGAGCCTGAAATTCTCCATTAAG	
OLLinker_Spe_fw	CCACTAGTATCAGCAGCAGGTGGTAAGCC	
OL_pHel2_Spe_inv	CCACTAGTGGATCCCCGGGTACCGAGC	
OLHP0103_1s	TAAAGATCTGTGCATTTAGAAGCTAAACTC	generation of pCJ513 and pCJ514 for unmarked *tlpB* mutant
OLHP0103_2s	ATAAGATCTGAAAGAATGGTGTCTTCAATGG	
OLHP0103_SpeI_3s	TATACTAGTGAGCATGATGAATGCTTCCATAG	
OLHP0103_SpeI_4s	TATACTAGTGAGATTTCATCGTTGCGATC	
OL_aphA3_X_F	TATTCTAGACGAACCATTTGAGGTGATAG	
OLHPFlaP_X_F	TATTCTAGATCATGCTCTTTTAAATTTTGC	
OLHP0103_5s	TATCCATGGATGATGTCAAATCCATTCTGG	
OLHP0103_6s	TATCCATGGATCGTCGCTGTCAGTATCC	
OLHP0099_1s	ATAAGATCTTAATGAGAACGATTGATCGTC	cloning of pCJ1396 for *tlpA* mutants (triple mutant)
OLHP0099_2s	AATAGATCTAGAGGTTATCCACTGAGCAGC	
OLHP0220_F2	TTTAGATCTGCCCATTATCATTACCGCTC	cloning of pCJ1350 for *nifSU* expression
OLHP0221_R	TTTAGATCTTCAAATCGGTAACACCCTGAT	

a: restriction sites underlined

Supplementary References

1. Behrens,W. *et al.* Role of energy sensor TlpD of *Helicobacter pylori* in gerbil colonization and genome analyses after adaptation in the gerbil. *Infect. Immun.* **81**, 3534-3551 (2013).
2. Sambrook,J. & Russel,D.G. *Molecular cloning: a laboratory manual.* 2004. Cold Spring Harbor, Cold Spring Harbor Laboratory Press.
3. Hanahan,D. Studies on transformation of *Escherichia coli* with plasmids. *J Mol Biol.* **166**, 557-580 (1983).
4. Casadaban,M.J. & Cohen,S.N. Analysis of gene control signals by DNA fusion and cloning in *Escherichia coli. J Mol Biol.* **138**, 179-207 (1980).
5. Schweinitzer,T. *et al.* Functional characterization and mutagenesis of the proposed behavioral sensor TlpD of *Helicobacter pylori. J. Bacteriol.* **190**, 3244-3255 (2008).
6. Copass,M., Grandi,G. & Rappuoli,R. Introduction of unmarked mutations in the *Helicobacter pylori vacA* gene with a sucrose sensitivity marker. *Infect. Immun.* **65**, 1949-1952 (1997).
7. Labigne-Roussel,A., Courcoux,P. & Tompkins,L. Gene disruption and replacement as a feasible approach for mutagenesis of Campylobacter jejuni. *J. Bacteriol.* **170**, 1704-1708 (1988).
8. Heuermann,D. & Haas,R. A stable shuttle vector system for efficient genetic complementation of *Helicobacter pylori* strains by transformation and conjugation. *Mol. Gen. Genet.* **257**, 519-528 (1998).
9. Cantwell,B.J. *et al.* CheZ phosphatase localizes to chemoreceptor patches via CheA-short. *J. Bacteriol.* **185**, 2354-2361 (2003).
10. Shevchenko,A., Tomas,H., Havlis,J., Olsen,J.V. & Mann,M. In-gel digestion for mass spectrometric characterization of proteins and proteomes. *Nat. Protoc.* **1**, 2856-2860 (2006).
11. Loewen,P.C. *et al.* Structure of *Helicobacter pylori* catalase, with and without formic acid bound, at 1.6 A resolution. *Biochemistry.* **43**, 3089-3103 (2004).
12. Abdiche,Y., Malashock,D., Pinkerton,A. & Pons,J. Determining kinetics and affinities of protein interactions using a parallel real-time label-free biosensor, the Octet. *Anal. Biochem.* **377**, 209-217 (2008).
13. Rust,M. *et al.* The *Helicobacter pylori* anti-sigma factor FlgM is predominantly cytoplasmic and cooperates with the flagellar basal body protein FlhA. *J Bacteriol.* **191**, 4824-4834 (2009).
14. Sterzenbach,T. *et al.* Role of the *Helicobacter hepaticus* flagellar sigma factor FliA in gene regulation and murine colonization. *J. Bacteriol.* **190**, 6398-6408 (2008).
15. Josenhans,C., Labigne,A. & Suerbaum,S. Comparative ultrastructural and functional studies of *Helicobacter pylori* and *Helicobacter mustelae* flagellin mutants: both flagellin subunits, FlaA and FlaB, are necessary for full motility in *Helicobacter* species. *J. Bacteriol.* **177**, 3010-3020 (1995).
16. Austin,C.M., Wang,G., & Maier,R.J. Aconitase functions as a pleiotropic posttranscriptional regulator in *Helicobacter pylori. J. Bacteriol.* **197**, 3076-3086 (2015).
17. Gruer,M.J., Bradbury,A.J., & Guest, J.R. Construction and properties of aconitase mutants of *Escherichia coli. Microbiology* **143,** 1837-1846 (1997).
18. Kennemann,L. *et al.* In Vivo Sequence Variation in HopZ, a Phase-Variable Outer Membrane Protein of *Helicobacter pylori. Infect. Immun.* **80,** 4364-4373 (2012).
19. Schmitz,A., Josenhans,C., & Suerbaum, S. Cloning and characterization of the *Helicobacter pylori flbA* gene, which codes for a membrane protein involved in coordinated expression of flagellar genes. *J. Bacteriol.* **179,** 987-997 (1997).
20. Schweinitzer, T., Mizote, T., Ishikawa, N., Dudnik, A., Inatsu, S., Schreiber, S. et al. Functional characterization and mutagenesis of the proposed behavioral sensor TlpD of *Helicobacter pylori. J. Bacteriol.* **190,** 3244-3255 (2008).
21. Schneider,C.A., Rasband,W.S. & Eliceiri,K.W. NIH Image to ImageJ: 25 years of image analysis. *Nat. Methods* **9**, 671-675 (2012).

Supplementary Figures

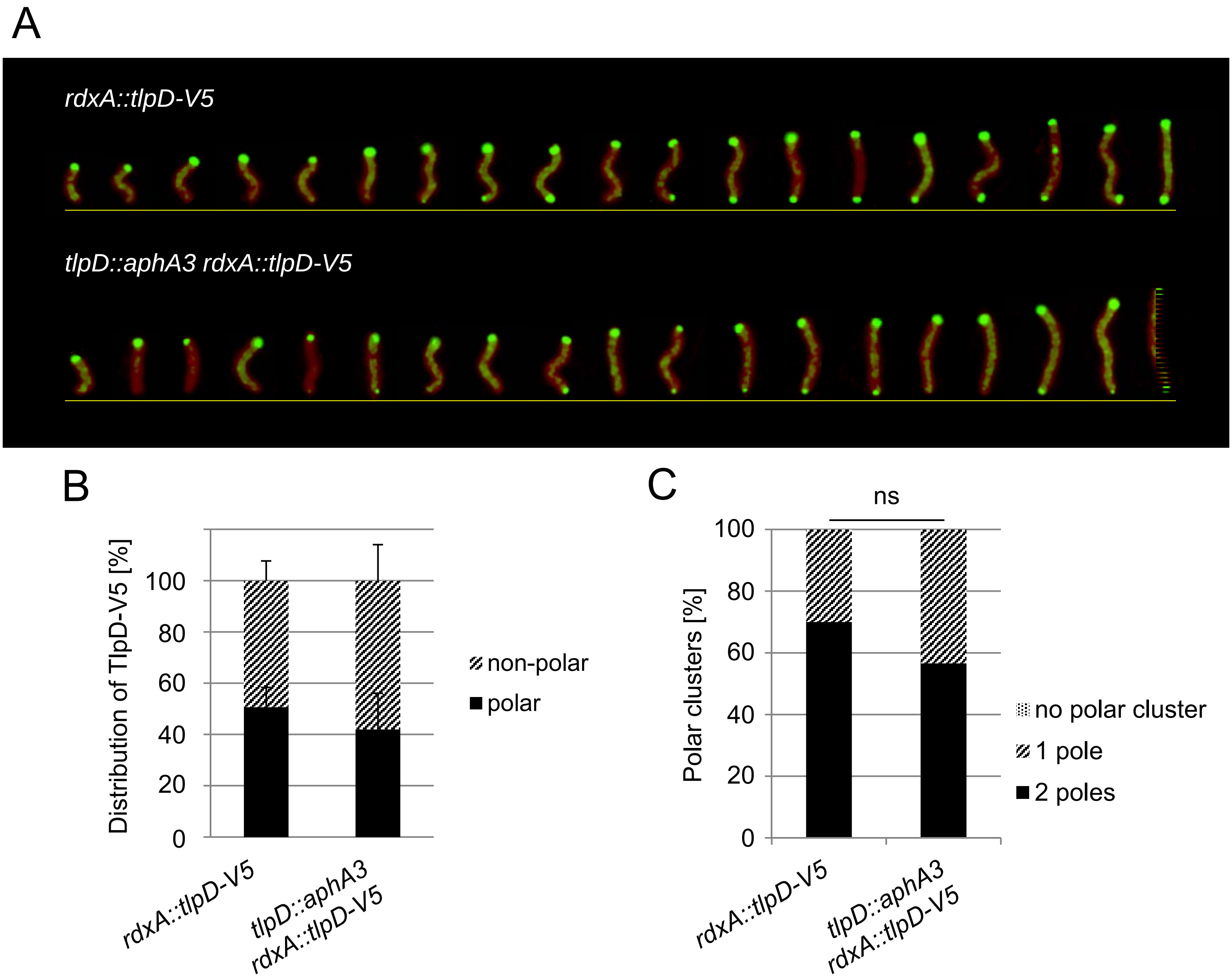

Fig. S1: Subcellular localisation of TlpD-V5 in *H. pylori* strains with and without expression of TlpD from the native locus.

(**A**) TlpD-V5 was imaged by immunfluorescence microscopy in intact *H. pylori* cells (strain N6) with (N6 *rdxA::tlpD-V5*) or without (N6 *tlpD::aphA3 rdxA::tlpD-V5*) the native chromosomal copy of untagged TlpD. The former strain carries two chromosomal copies of TlpD in different genomic locations, while the latter strain was used as a control strain which has only one copy of TlpD (V5-tagged). Green: TlpD-V5, detected using α-V5 antibody (primary, mouse, at 1:1,000 dilution), combined with α-mouse IgG (coupled to Alexa-488, at 1:5,000); Red: SynaptoRed FM4-64 (1:5,000) membrane-intercalating dye as counterstain. All samples were prepared from 20 h plate-grown bacteria directly resuspended in fixing agent. (**B**) The subcellular distribution of TlpD-V5 (fluorescence intensity) in both strains was first quantitated as sum of pixel intensities along the longitudinal axis and then subdivided into polar and non-polar pixel intensities as percent of total intensities per strain (averaged over 30 bacteria per strain, Supplementary Methods). Statistical differences between both strains were calculated to be non-significant (Student's *t* test), except for the comparison of the polar intensity, which was moderately but significantly higher for the N6 *rdxA::tlpD-V5* strain. (**C**) Quantification of polar TlpD clusters per cell for ≥ 30 bacteria per strain (in percent of evaluated bacteria of each strain). Levels of significance between the two strains were calculated by Fisher's exact test for clusters at 1 vs. 2 poles. ns: not significant. Four separate parameters: mean total TlpD-V5 fluorescence intensity, mean non-polar intensity, mean non-polar intensity distribution of TlpD-V5, and occurrence and distribution of polar TlpD clusters, that were all averaged over 30 bacteria, were not significantly different between N6 *rdxA::tlpD-V5* and the control strain N6 *tlpD::aphA3 rdxA::tlpD-V5* (Student's *t* test and Fisher's exact test). Densitometry of Western blots of soluble and insoluble fractions also showed only minor differences in the distribution of TlpD-V5 between the two strains (not shown).

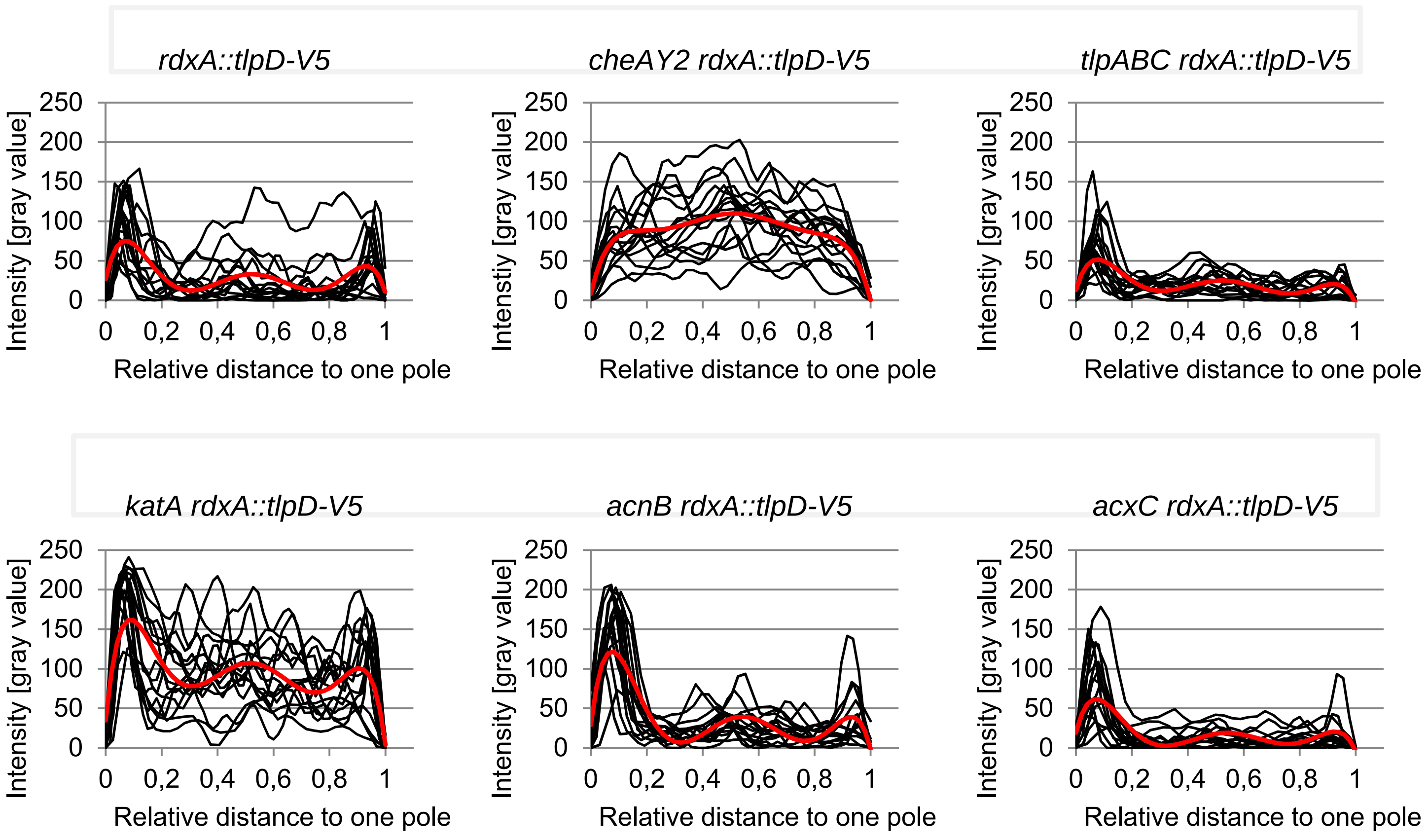

Fig. S2: Longitudinal intensity profiles from TlpD-V5-derived fluorescence signal of plate-grown *H. pylori*.

Black lines: TlpD-V5 intensity profiles (immunofluorescence, see Suppplementary Methods and Fig. S1) along the longitudinal axis as overlay of 15 single bacteria per strain. Cell length was set to a constant length of 1 to compare bacteria of different length. An approximation of every single profile independently of the individual cell length was then determined by a trend line of polynomial type (maximal exponent of 6). To depict an average intensity distribution for every strain, an average curve was calculated from all trend lines of one strain. The average trend line is highlighted in red (see also Material and Methods for description of calculations).

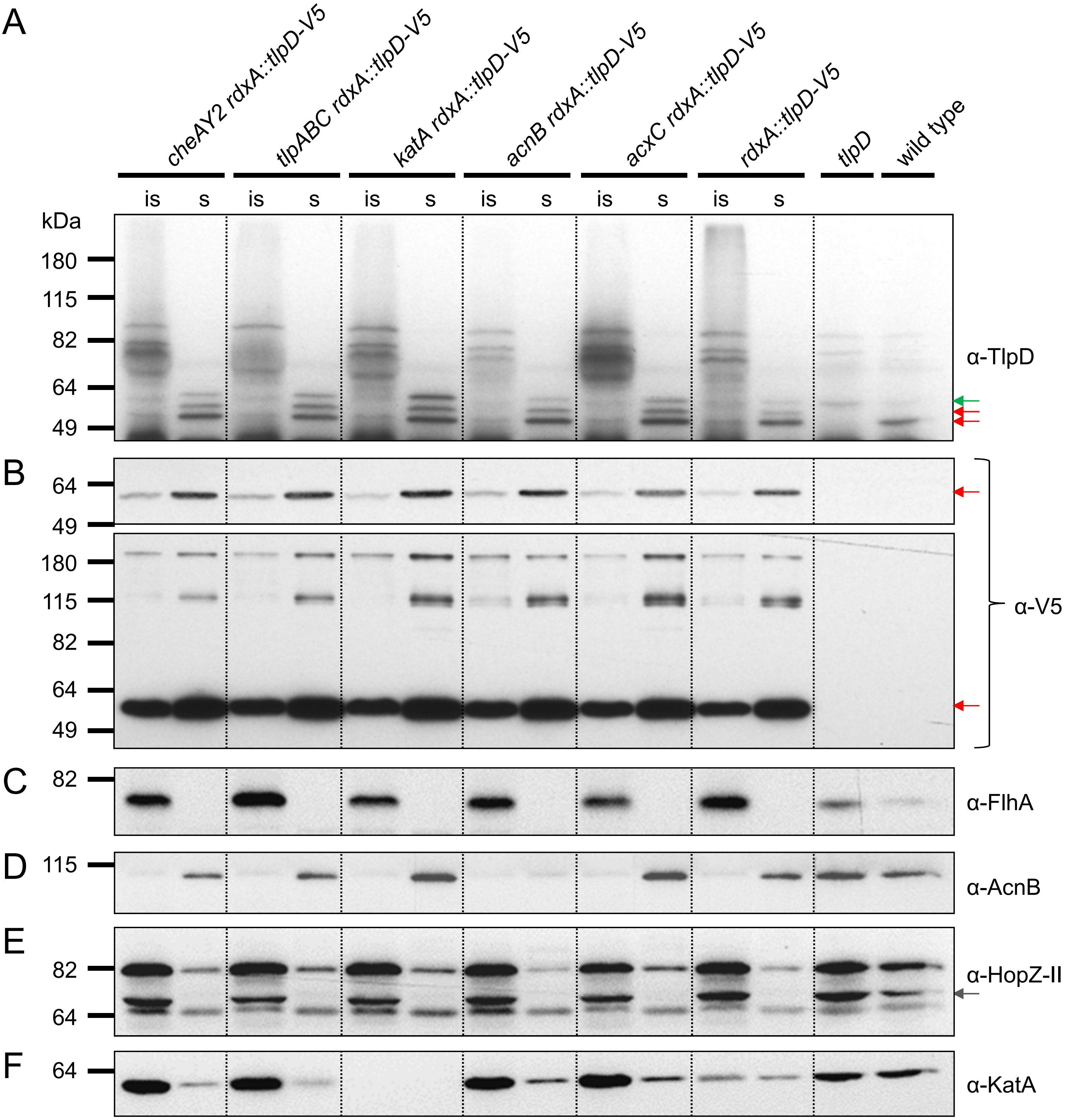

Fig. S3: Localisation of *H. pylori* TlpD in fractionated cell lysates of parental strain N6 and its TlpD interactor mutants.

Detection of TlpD in crude fractions (insoluble, soluble) of cell lysates of *H. pylori strain* N6 expressing TlpD or TlpD-V5 (chromosomal integration) and respective mutants in the same strain lacking potential TlpD interaction partners (see Supplemental Methods for crude fractionation and detection of proteins). Total lysates of *tlpD* mutant and N6 parental strain were used as controls. (**A**) TlpD was detected using α-TlpD or (**B**) α-V5 antisera in Western blot (red arrows point at monomeric TlpD and TlpD-V5 respectively). The green arrow in panel (**A**) points at a non-specific band. Fractionation controls were performed using (**C**) α-FlhA (membrane-associated), (**D**) α-AcnB (cytoplasmic), (**E**) α-HopZ-II (outer membrane, grey arrow), (**F**) α-KatA antisera. is: insoluble cell fraction (membrane-enriched), s: soluble cell fraction. Quantitation of TlpD protein band intensities was performed with ImageJ and normalised against common protein bands detected with antiserum against whole *H. pylori* bacteria. Quantification of TlpD in the reference strain showed a distribution of about 5% and 95% in the insoluble and soluble fraction, respectively. Total quantity and fraction distribution of TlpD were not significantly different between the different strains as determined by chi-square test and differed maximally by 9%.

Behrens *et al*., Supplementary Fig. S3

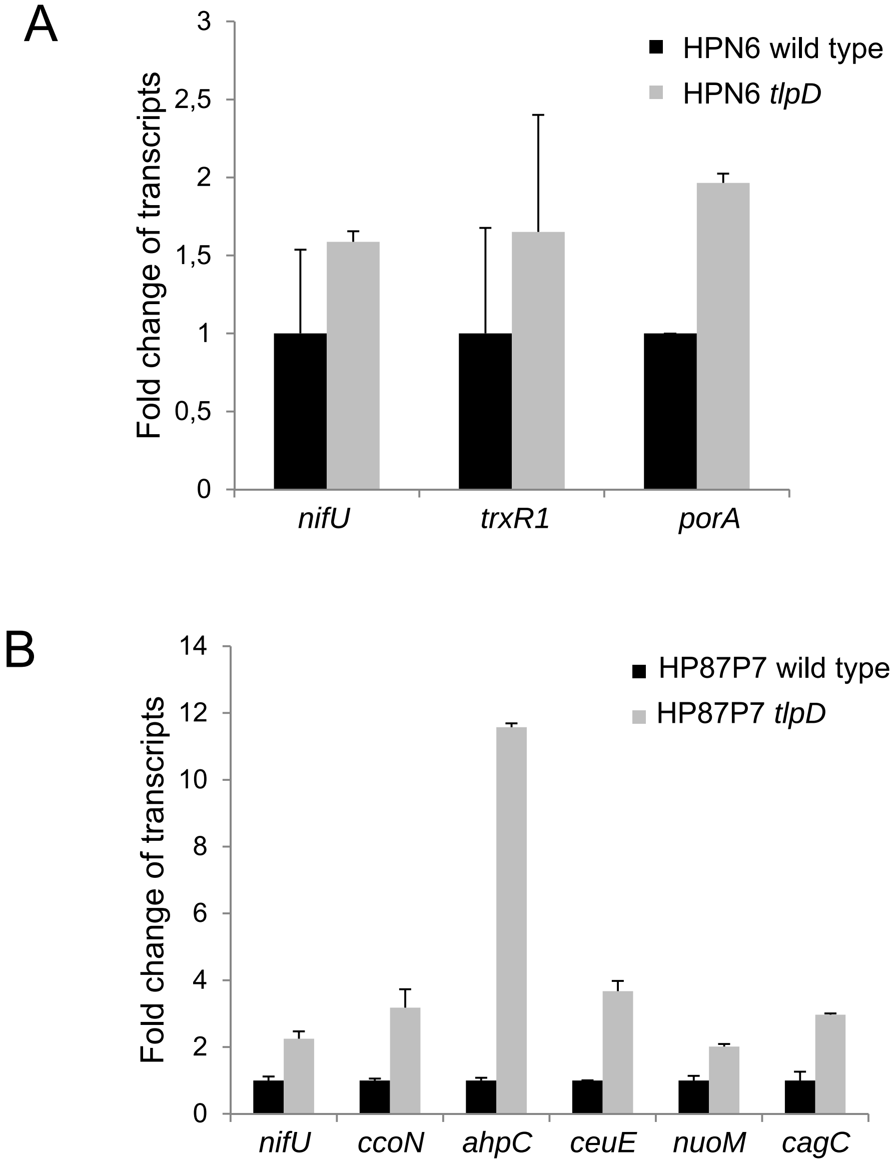

Fig. S4: Relative transcript amounts of genes in *H. pylori tlpD* mutants vs. wild type determined by qPCR.

Transcript amounts for several genes derived from transcriptome analyses (see main text) were determined by qPCR using cDNA from (A) strain N6 and (B) strain HP87P7 (see Supplemental Methods for RNA preparation and qPCR). The depicted genes showed the following mean ratios (*tlpD* versus wild type) in four microarrays performed for strain N6: *nifU*: 1.89, *trxR1*: 1.75, *porA*: 1.52, *ccoN*: 1.61, *ahpC*: 1.89, *ceuE*: 1.16, *nuoM*: 1.08, *cagC*: 2.17. Except for *cagC*, the genes are not included in Supplementary Tables S2 and S3, since their mean ratios were below the cut-off of two-fold regulated. In single biological array experiments, these genes showed more than two-fold altered transcript amounts.

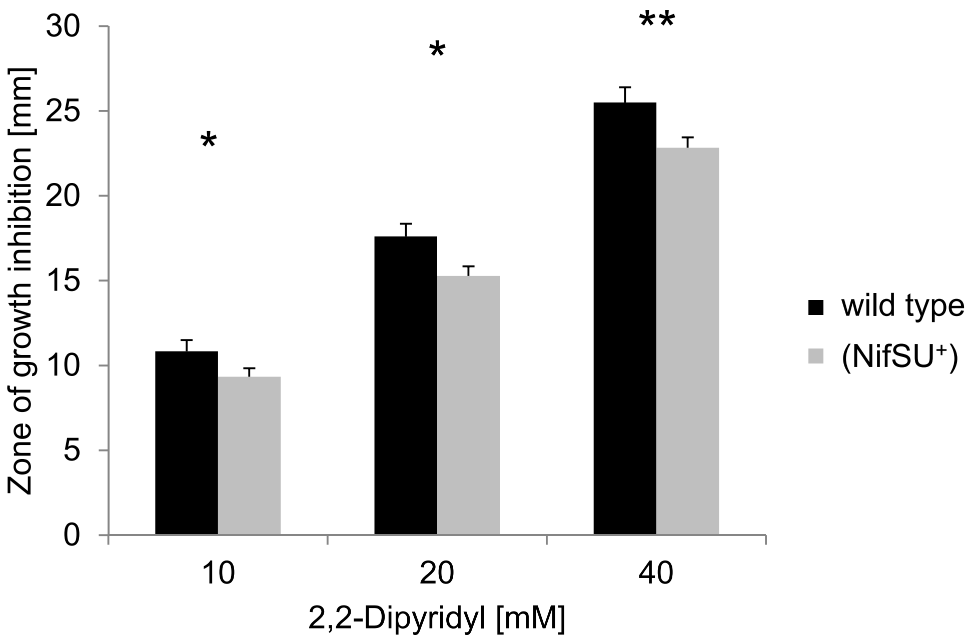

Fig. S5: Growth inhibition of *H. pylori* wild type and NifSU overexpression strain under conditions of iron-depletion.

Defined numbers of bacteria (strain N6) were plated on BHI/yeast + 5% horse serum plates and incubated with paper discs soaked with 10 µl 10, 20 and 40 mM 2,2-dipyridyl for iron depletion. The zones of growth inhibition around the discs were determined at 72 h of incubation. Results from one representative experiment (technical triplicates) are depicted. Significance levels were calculated with Student's *t* test (* $p<0.05$; ** $p<0.01$). Inhibition zones of the $NifSU^+$ strain are smaller than those of the parent under these assay conditions, indicating that the $NifSU^+$ strain is less susceptible to iron depletion.

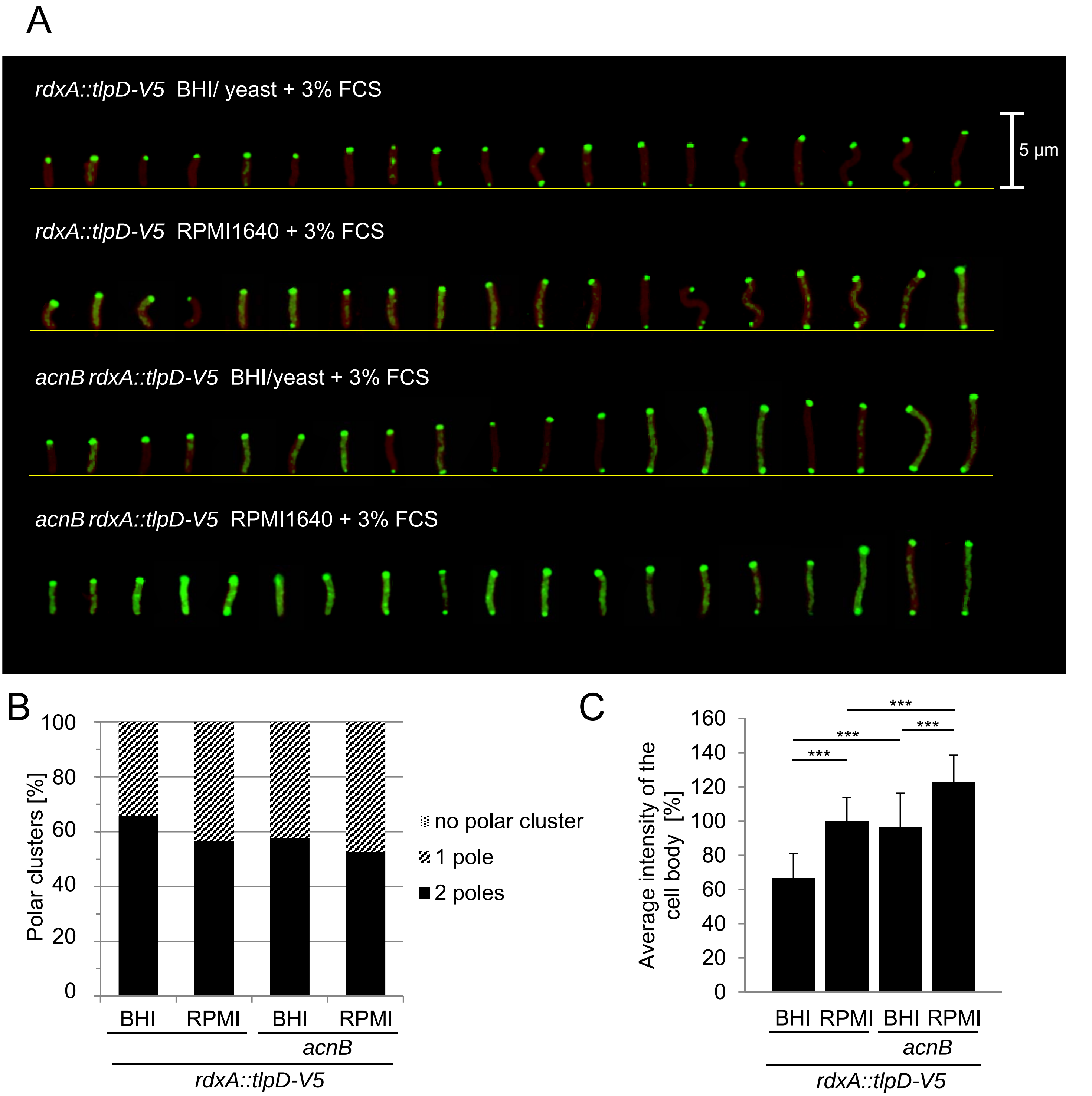

Fig. S6: Control fluorescence data for main Fig. 6, addressing TlpD subcellular localisation under different energetic conditions (exemplary for the reference strain N6 *rdxA::tlpD-V5* and the corresponding *acnB* mutant). (A) Fluorescent images of TlpD-V5 in both strains incubated in two media of different energy yield. TlpD-V5 (green) was detected by α-V5 MAB (1:1,000) in strain N6 *rdxA::tlpD-V5* and the corresponding *acnB* mutant after 20 min incubation in two liquid media, BHI/yeast + 3% fetal calf serum (FCS; high energy) or RPMI1640 + 3% FCS (low energy). The media differ by their nutrient levels and energy yield in *H. pylori*[1]. Bacteria were fixed immediately after the incubation time. Red: bacterial membranes counterstained with SynaptoRed FM4-64 (1:5,000). **(B) Fluorescence quantitation control detecting non-significant differences of TlpD-V5 polar localisation in *H. pylori* upon incubation in liquid media of divergent energy yield.** The presence and distribution of polar clusters in both strains was counted and quantitated in at least 30 bacteria per condition (Methods) and was determined not to be statistically different between strains and conditions. Distribution is indicated in percent of all bacteria counted for each strain and condition. **(C) TlpD quantitation in the bacterial cell body of *acnB* mutant and parental strain suggest an influence of AcnB on TlpD localisation.** Fluorescence intensities of TlpD-V5 along the bacterial cell body omitting the polar regions (see Methods and legend to main Figure 6) were quantitated in N6 *rdxA::tlpD-V5* (reference) and the corresponding *acnB* mutant upon incubation in different media. For each condition, 30 bacteria were measured. The average intensity of the cell body of the reference strain in RPMI medium was set to 100%; the other values are depicted as relative values in percent. Mean values and standard deviations are shown. Significance of differences was determined by Student's *t* test (*** $p<0.001$).

Behrens *et al.*, Supplementary Fig. S6
